# Characterization of the Far Transcription Factor Family in *Aspergillus flavus*

**DOI:** 10.1534/g3.116.032466

**Published:** 2016-08-16

**Authors:** Xingyu Luo, Katharyn J. Affeldt, Nancy P. Keller

**Affiliations:** * Department of Plant Pathology, University of Wisconsin-Madison, Wisconsin 53706; † Department of Medical Microbiology and Immunology, University of Wisconsin-Madison, Wisconsin 53706; ‡ Department of Bacteriology, University of Wisconsin-Madison, Wisconsin 53706

**Keywords:** fatty acid metabolism, aflatoxin, virulence, β-oxidation, gene family, FlyBook

## Abstract

Metabolism of fatty acids is a critical requirement for the pathogenesis of oil seed pathogens including the fungus *Aspergillus flavus*. Previous studies have correlated decreased ability to grow on fatty acids with reduced virulence of this fungus on host seed. Two fatty acid metabolism regulatory transcription factors, FarA and FarB, have been described in other filamentous fungi. Unexpectedly, we find *A. flavus* possesses three Far homologs, FarA, FarB, and FarC, with FarA and FarC showing a greater protein similarity to each other than FarB. *farA* and *farB* are located in regions of colinearity in all *Aspergillus* spp. sequenced to date, whereas *farC* is limited to a subset of species where it is inserted in an otherwise colinear region in *Aspergillus* genomes. Deletion and overexpression (OE) of *farA* and *farB*, but not *farC*, yielded mutants with aberrant growth patterns on specific fatty acids as well as altered expression of genes involved in fatty acid metabolism. Marked differences included significant growth defects of both ∆*farA* and ∆*farB* on medium-chain fatty acids and decreased growth of *OE*::*farA* on unsaturated fatty acids. Loss of *farA* diminished expression of mitochondrial β-oxidation genes whereas *OE*::*farA* inhibited expression of genes involved in unsaturated fatty acid catabolism. FarA also positively regulated the desaturase genes required to generate polyunsaturated fatty acids. Aflatoxin production on toxin-inducing media was significantly decreased in the ∆*farB* mutant and increased in the *OE*::*farB* mutant, with gene expression data supporting a role for FarB in tying β-oxidation processes with aflatoxin accumulation.

*Aspergillus flavus* is an opportunistic plant pathogen that causes disease both pre- and postharvest on oil-rich seed crops, such as maize, peanuts, tree nuts, and cottonseed (Amare and Keller 2014). Even within these seeds, *A. flavus* primarily colonizes the lipid-rich portions (Brown *et al.* 1995; Keller *et al.* 1994). Upon infection of its host, *A. flavus* synthesizes the polyketide secondary metabolite aflatoxin (AF). AF is both toxic and carcinogenic leading to international allowances of acceptable amounts of AF in food and feeds; consequently, there is great interest in identifying the virulence factors of *A. flavus*.

Several studies have demonstrated a correlation between virulence, AF production, and ability to metabolize lipids in *A. flavus*. For example, LaeA, a putative methyltransferase and global regulator of many secondary metabolite clusters in *A. flavus*, including the AF cluster (Georgianna *et al.* 2010), is impaired in seed colonization in part due to reduced lipase activity (Kale *et al.* 2008). LaeA interacts with another protein, VeA (Bayram *et al.* 2008; Chang *et al.* 2013), which is also required for virulence and AF production on seed. Both ∆*laeA* and ∆*veA* exhibit diminished utilization of host lipids *in planta* as well as increased sensitivity to oleic acid *in vitro* (Amaike and Keller 2009). The oxylipin-producing Ppo oxygenases impact virulence in both *A. flavus* and *A. nidulans*. *A. nidulans* ∆*ppoB* displays hypervirulence and enhanced lipase activity, while ∆*ppoA*;∆*ppoC* and ∆*ppoA*;∆*ppoB*;∆*ppoC* double and triple mutants are attenuated in virulence and lipase activity (Tsitsigiannis and Keller 2006; Brown *et al.* 2009). Both the type and concentration of fatty acids and their respective oxylipins affect AF synthesis (Yan *et al.* 2015; Scarpari *et al.* 2014) and loss of a caleosin-like protein affects oxylipin synthesis, resulting in reduced virulence and decreased AF production (Hanano *et al.* 2015). Taken together, these data suggest that lipid metabolism is an important component of seed infection by *A. flavus* and possibly other *Aspergillus* species.

A pair of conserved Zn2-Cys6 transcription factors, CTF1α and CTF1β, were first described in *Fusarium solani* f. sp. *pisi* and found to regulate fatty acid metabolism genes. CTF1α and CTF1β regulate cutinase gene expression by binding to a conserved CCTCGG motif found upstream of those genes (Li and Kolattukudy 1997; Li *et al.* 2002). Orthologs of CTF1α and CTF1β were then described in *A. nidulans* and named *farA* and *farB*, respectively. Characterization of *farA* and *farB* showed that they regulate genes involved in fatty acid metabolism, also via binding to a CCTCGG motif (Hynes *et al.* 2006). Ctf/Far transcription factors are important for virulence in both animal and plant hosts. They are conserved in other fungi and have been characterized to some degree in *A. oryzae*, *Candida albicans*, *F. oxysporum* f. sp. *lycopersici*, and *Yarrowia lipolytica* (Bravo-Ruiz *et al.* 2013; Garrido *et al.* 2012; Poopanitpan *et al.* 2010; Ramírez and Lorenz 2009; Rocha *et al.* 2008).

The work in *A. nidulans* showed *farA* to be required for normal growth on any length of fatty acid, while *farB* was only needed for growth on short-chain fatty acids (Hynes *et al.* 2006). Proliferation of peroxisomes is dependent on PexK (Pex11), the expression of which is activated by the FarA transcription factor. Proliferation was greatly reduced in a *farA* deletion strain and thought to contribute to the growth defect on fatty acids (Hynes *et al.* 2008). In *A. oryzae*, whose genome is nearly identical to that of *A. flavus*, the ∆*farA* mutant was unaffected in its ability to grow on short-chain fatty acids, and it only exhibited a slight reduction in growth on medium- and long-chain fatty acids. Although *A. oryzae* possesses *farB*, this gene was not examined (Garrido *et al.* 2012).

Here, we investigated the role of Far homologs in *A. flavus* fatty acid metabolism and virulence on host seed. Unexpectedly, we found that *A. flavus* contains three Far homologs: FarA, FarB, and FarC. An examination of the sequenced Aspergilli showed that whereas *farA* and *farB* were present in all species in a region of colinearity of the genome, *farC* was only present is a subset of species and likely represented a pseudogene whose predicted product lacks a canonical DNA binding motif. Expression analysis and examination of gene disruption and overexpression mutants showed both FarA and FarB, but not FarC, to be involved in various aspects of fatty acid metabolism, with FarB playing a possible activation role in AF biosynthesis, and FarA likely involved in mitochrondrial β-oxidation and unsaturated fatty acid catabolism. Both FarA and FarB also demonstrate a regulatory role in the expression of genes required for unsaturated fatty acid biosynthesis.

## Materials and Methods

### Culture conditions

Strains (Supplemental Material, Table S1) were grown on glucose minimal medium (GMM) (Shimizu and Keller 2001) with 70.6 mM ammonium chloride as the nitrogen source. In some cases, 5 mM uridine and 5 mM uracil were added, and this is denoted “+ UU.” For genomic DNA extraction, strains were grown on liquid GMM (NH_4_^+^) with 0.5% yeast extract added.

### Strain construction

Strain genotypes and sources are summarized in Table S1. All primers used for strain construction are listed in Table S2. *farA*, *farB*, and *farC* were disrupted using homologous recombination to replace each gene with *A. fumigatus pyrG* in the parental strain CA14∆*ku70*∆*pyrG* (Chang *et al.* 2010). The *farA* 5′ and 3′ flanks were amplified with primers 1 and 2 and primers 3 and 4, respectively. The *farB* 5′ and 3′ flanks were amplified with primers 5 and 6 and primers 7 and 8, respectively. The *farC* 5′ and 3′ flanks were amplified with primers 9 and 10 and primers 11 and 12, respectively. *A. fumigatus pyrG* was amplified from genomic DNA using primers 13 and 14. Primers 1 and 4, primers 5 and 8, and primers 9 and 12 were used to amplify the entire disruption constructs for *farA*, *farB*, and *farC*, respectively.

*farA*, *farB*, and *farC* were each overexpressed at their native loci using double-joint PCR constructs. Overexpression was driven by the *A. nidulans gpdA* promoter, and the marker gene was *A. parasiticus pyrG*. The *gpdA* promoter was inserted immediately upstream of the ATG start site. The *pyrG*::*gpdA(p)* fragment was amplified from plasmid pJMP9.1 (J. M. Palmer and N. P. Keller, unpublished results) using primers 15 and 16. The *farA* 5′ flank was amplified with primers 17 and 18, and the first ∼1 kb of the *farA* coding region was amplified with primers 19 and 20 and placed at the 3′ end of the double-joint construct. The primers used to amplify the *OE*::*farB* 5′ flank were 21 and 22, and the primers for the first ∼1 kb of the *farB* coding region were 23 and 24. The primers used to amplify the *OE*::*farC* 5′ flank were 9 and 25, and the primers for the first ∼1 kb of the *farC* coding region were 26 and 27. Primers 17 and 20, primers 21 and 24, and primers 9 and 27 were used to amplify the entire overexpression constructs for *farA*, *farB*, and *farC*, respectively.

All of the above constructs were transformed individually into parental strain CA14∆*ku70*∆*pyrG*. Transformation of the fungus was carried out according to the protocol of Szewczyk *et al.* (2006) with the following modifications: 200 mg of *Trichoderma* lysing enzymes (Sigma-Aldrich) were added to 10 ml KCl protoplasting solution, and the protoplasts were plated on sorbitol minimal medium (SMM) [GMM (NH_4_^+^) plus 1.2 M sorbitol]. All strains were confirmed by PCR, Southern analysis, and, in the case of the overexpression mutants, semiquantitative RT-PCR. The primers used to make the ∆*farA* and ∆*farB* Southern probes were primers 1 and 4, and 5 and 8, respectively. The primers used to make ∆*farC* Southern probes were primers 9 and 10, and primers 11 and 12. The primers used for the *OE*::*farA* Southern probe were primers 17 and 18 and primers 19 and 20, and the primers used for the *OE*::*farB* Southern probe were primers 21 and 24. The primers used for the *OE*::*farC* Southern probe were primers 9 and 25 and primers 26 and 27.

### Sequence, phylogenetic, and colinearity analysis

Protein sequences obtained from Genbank (http://www.ncbi.nlm.nih.gov/) and AspGD (http://www.aspgd.org) were aligned using MUSCLE. Conserved regions of the alignment were sampled, and a maximium likelihood phylogenetic tree (topology only) were created using *MEGA* version 6.0 with 100 bootstraps. The colinearity analysis was done on AspGD website http://www.aspergillusgenome.org.

### Pathogenicity assays

Pathogenicity assays were conducted according to the published protocol (Christensen *et al.* 2012) with some modifications. Kernels were washed in 70% EtOH for 5 min, rinsed with sterile water, and shaken in bleach (100 rpm) for 10 min. After three rinses in sterile water to remove the bleach, the kernels were blotted dry on sterile paper towels and a sterile needle was used to puncture a small hole in the embryo of each kernel. Four kernels were placed into a sterile scintillation vial, weighed, and inoculated with 200 μl of a 10^6^ spore/ml suspension of spores in 0.01% Tween 20. A mock control inoculated with just 0.01% Tween 20 was also included. The kernels were adjusted with a sterile spatula so that the embryo was always facing up, and vials were placed in a plastic box containing moist paper towels. The box was covered with Press’n Seal (Glad) and placed in a 29° incubator with 12 hr light/dark cycles (beginning on dark) for 3 and 5 d. Four replicates per strain per time point were included.

Following incubation, the kernels were vortexed in 2.5 ml 100% MeOH to remove the spores. A 100 μl aliquot of spores was removed, diluted, and counted on a hemocytometer. A total of 5 ml of chloroform was added to the vials, and they were incubated in the dark overnight to extract ergosterol and aflatoxin. 1 ml of the extract was removed for AF analysis. It was dried down, resuspended in 1 ml 50:40:10 water:methanol:acetonitrile, filtered through an Acrodisc syringe filter with nylon membrane (0.45 μm, Pall Corporation), and run on a Perkin Elmer Flexar Instrument equipped with a ZORBAX Eclipse XDB-C18 column (150 × 4.6 mm, 5 μm, and 100 Å, Agilent). The column was equilibrated in the running solvent (50:40:10 water:methanol:acetonitrile), and an isocratic method was run for 11 min with 100% running solvent at a flow rate of 0.8 ml/min. The column was reequilibrated in 100% running solvent for 30 sec prior to each injection (20 μl). AF was detected by a fluorescent detector with an emission wavelength of 455 nm and excitation wavelength of 365 nm.

The remaining portion of the original extract was dried down and resuspended in 1 ml water and 3 ml heptane. Then, 2 ml were removed from the heptane layer, dried down, resuspended in 1 ml methanol, and filtered as above. This was run on the same HPLC system as described for AF with the following isocratic method: 100% methanol was flowed at a rate of 1.5 ml/min for 8 min per run. The column was reequilibrated in 100% methanol for 30 sec prior to each injection (50 μl). Ergosterol was detected by a PDA detector set to 282 nm with a reference wavelength of 400 nm.

### Aflatoxin extraction and analysis

For AF production on solid YES medium, strains were grown on 10 cm diameter plates containing 25 ml YES (2% yeast extract and 6% sucrose, pH 5.8) or YEP (2% yeast extract and 6% peptone, pH 5.8) with uridine and uracil (+ UU) and 1.5% agar. Then, 10^3^ spores in 1 μl of 0.1% Tween 20 were point inoculated onto the center of each plate. Three replicates per strain were placed in the dark at 29° for 5 d. A 15 mm diameter core was punched from each plate and homogenized in 3 ml 0.01% Tween 20.

To extract AF, 3 ml of ethyl acetate were added to each tube, and the tubes were shaken vigorously and spun at 3000 rpm for 10 min. The organic layer was removed, dried down, and resuspended in 1 ml 50:40:10 water:methanol:acetonitrile, and HPLC analysis was carried out as described above.

For AF production on liquid YES medium, 10^6^ spores/ml of each strain were inoculated into 50 ml YES + U/U in 125 ml flasks. Three replicates per strain were shaken in the dark at 29° and 250 rpm for 36 and 48 hr, respectively.

To extract AF, 25 ml of chloroform were added to each flask, and the flasks were shaken vigorously. Then, 10 ml of the organic layer was removed, dried down, and resuspended in 1 ml 50:40:10 water:methanol:acetonitrile, and HPLC analysis was carried out as described above.

### Sensitivity to carbon sources of varying chain length

To assess the roles of FarA, FarBl, and FarC in sensitivity to various carbon sources, strains were grown on a variety of media as in Hynes *et al.* (2006) with some modifications. The following carbon sources were added to minimal medium with uridine and uracil and 70.6 mM ammonium chloride as the nitrogen source: 1% glucose, 50 mM sodium acetate (C2), 50 mM propionic acid (C3), 10 mM sodium butyrate (C4), 2.5 mM lauric acid (C12), 2.5 mM myristic acid (C14), 2.5 mM palmitic acid (C16:0), 2.5 mM margaric acid (C17), 2.5 mM stearic acid (C18:0), 2.5 mM oleic acid (C18:1), 2.5 mM linoleic acid (C18:2), 2.5 mM erucic acid (C22:1), 0.1% Tween 20, 0.1% Tween 80, and 1% corn oil. Long-chain fatty acids were dispersed in media by the addition of 0.5% tergitol (NP-40, Sigma, St. Louis) after autoclaving. 40 ml of media were dispensed into square 10 cm × 10 cm plates and inoculated with 10^3^ spores of each strain in 1 μl 0.1% Tween 20. Plates were incubated at 29° for 3 d, and there were three replicate plates per condition.

### RNA extraction and analysis

Gene expression was examined in *far* mutants growing on a variety of fatty acids and glucose in both liquid and solid culture. For liquid culture, 10^6^ spores/ml of each strain were inoculated into 50 ml GMM (NH_4_^+^) + UU in 125 ml flasks. Flasks were shaken at 200 rpm at 29° for 24 hr. Mycelia were collected, washed, and transferred into fresh flasks. The second set of flasks contained minimal medium (UU + 1% tergitol + 70.6 mM ammonium chloride) with 1% glucose, 50 mM acetic acid (C2), 50 mM propionic acid (C3), 10 mM sodium butyrate acid (C4), 2.5 mM myristic acid (C14), 2.5 mM palmitic acid (C16:0), 2.5 mM stearic acid (C18:0), 2.5 mM oleic acid (C18:1), and 2.5 mM linoleic acid (C18:2). Cultures were shaken as before for 1 or 4 hr, respectively. After the second incubation, mycelia were collected, washed, and lyophilized. For solid culture, 10^3^ spores of each strain in 1 μl 0.1% Tween 20 were inoculated onto minimal media amended with either glucose, propionic acid (C3), myristic acid (C14), (C18:0), oleic acid (C18:1), or linoleic acid (C18:2), respectively. The media compositions were the same as described in the fatty acid growth test above. The media surface was covered by cellophane sheets prior to inoculation. Plates were incubated at 29° for 3 d. After the incubation, mycelia were peeled off the cellophane sheets and lyophilized.

To evaluate expression of a variety of genes (listed in Table S3) on *far* mutants growing on YES medium, 10^6^ spores/ml of each strain were inoculated into 50 ml YES + UU in 125 ml flasks. Flasks were shaken at 250 rpm at 29° for 36 and 48 hr, respectively. Mycelia were collected, washed, and lyophilized.

RNA was extracted using QIAzol Lysis Reagent (Qiagen). 10 μg of RNA was treated with DNase I (New England BioLabs) and used as the template for cDNA synthesis using the iScriptTM cDNA Synthesis kit (Bio-Rad). A total of 50 ng cDNA was used as template DNA for semiquantitative PCR reactions. The optimal annealing temperature was determined by PCR of genomic DNA (gDNA), and a sample of gDNA was always included as a positive control. The number of amplification cycles was optimized for each primer set as listed in Table S3, as are all the primers used for semiquantitative PCR. The entire 25 μl PCR reaction was loaded into a 1.5% agarose gel containing ethidium bromide, separated by gel electrophoresis, and imaged under UV light. The intensities of the bands were quantified using Adobe Photoshop, and imaged expression intensity for each gene was normalized to the expression intensity of the gene encoding ubiquitin, *ubiD*, for each sample.

### Statistical analysis

Statistically significant differences were determined by an unpaired Student’s *t*-test with a two-tailed distribution and *P* < 0.05. The error bars in all figures indicate the SD of the data set.

### Data availability

All strains are listed in Table S1 and are available upon request. 

## Results

### Identification of three Far homologs in A. flavus

**Figure 1:**
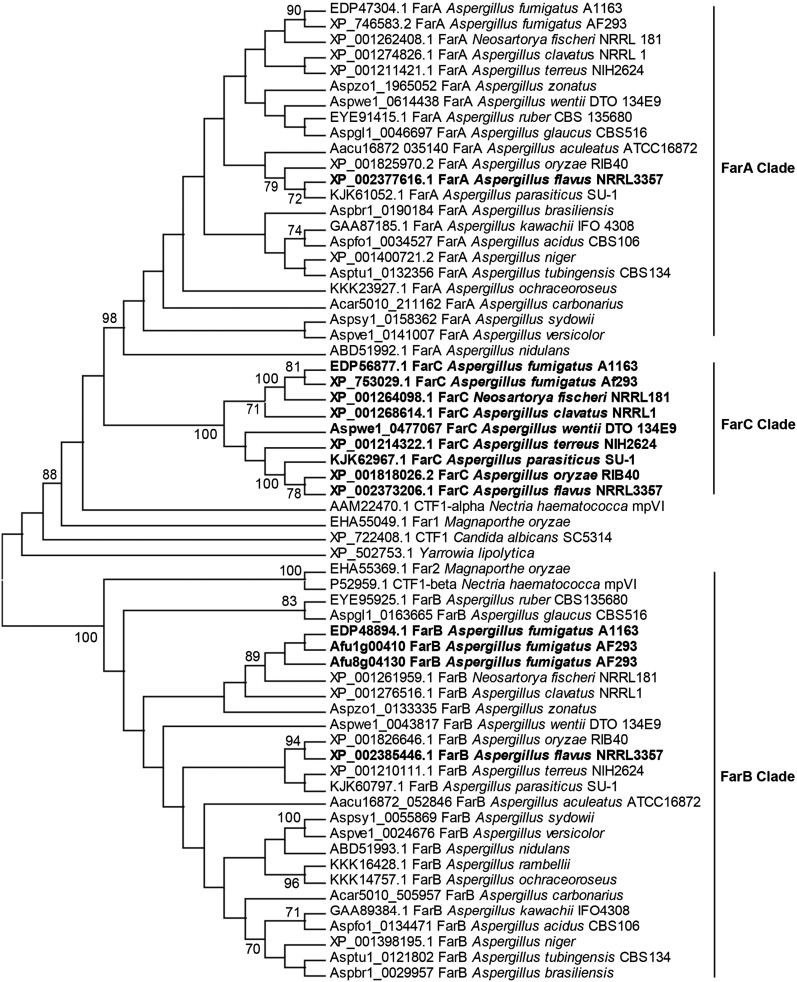
Phylogenetic analysis of amino acid sequences of three Far proteins. Maximum likelihood phylogenetic tree (topology only) was created using *MEGA* version 6.0 (Tamura *et al.* 2013). Bootstrap values above 70% of the 100 performed reiterations are shown. Bold texts indicate species of interest.

Three possible homologs of *A. nidulans* FarA (AN7050 ) and FarB (AN1425 ) were identified in a BLASTp search of the *A. flavus* genome. Two of these homologs (*farA*, AFL2G_05109 and *farB*, AFLA_012010 ) were located in regions of colinearity with *far* genes in other sequenced *Aspergillus* species. One interesting observation was that farB and its surrounding genes have an isolated duplication in the *A. fumigatus* AF293 strain that is not present in the *A. fumigatus* A1163 strain (Figure 1 and data not shown). However, *farC* (AFLA_082910 ) was found only in a subset of *Aspergillus* species where it was inserted in a region of colinearity between two genes, one encoding a short-chain dehydrogenase and the other a SWR-1 complex protein (Figure S1). Both *A. flavus* FarA and FarB are composed of a Zn2-Cys6 DNA binding domain and a fungal-specific transcription factor domain, while all *Aspergillus* FarC orthologs possess only the regulatory domain (Figure S2).

A phylogenetic analysis of Far proteins revealed that, among *Aspergillus* spp., FarC proteins were more closely related to FarA than FarB. The tree shows a FarA clade that includes FarA from *Aspergillus* spp., Far1 from *Magnaporthe oryzae*, and CTF1α from *Nectria hematococca*; a FarB clade that includes FarB from *Aspergillus* spp, Far2 from *M. oryzae*, and CTF1β from *N. hematococca*; and a FarC clade that includes FarC from *Aspergillus* species. The FarC clade, comprising only *Aspergillus* proteins, is found within the FarA clade and is more closely related to *Aspergillus* FarA orthologs than the *Sordariomycete* FarA orthologs. POR1 from the yeast *Y. lipolytica* and CTF1 from *C. albicans* were basal to FarA and FarB (Figure 1).

### Deletion and overexpression of farA, farB, and farC

To characterize *farA*, *farB*, and *farC* in *A. flavus*, all three genes were individually deleted and overexpressed. The sequence of the *A. flavus far* genes were compared from both Genbank (NCBI) (http://www.ncbi.nlm.nih.gov/) and AspGD (http://www.aspergillusgenome.org). We found the two sequences differed (Figure S3) and, based on comparison with other known *far* genes, it appeared that both *farA* and *farB* were misannotated in Genbank; furthermore, the introns of *farA* were misannotated in AspGD. To correct this, we assessed intron predictions of *A. oryzae farA* to *A. flavus farA* and obtained a gene sequence whose encoded protein contained a consensus amino acid sequence of C6 finger transcription factor (Figure S3). *farC* also appeared to be misannotated in the *Aspergillus* genome site (AspGD) (http://www.aspergillusgenome.org) for several species including *A. flavus*. The final best prediction of each protein yielded a 903 amino acid FarA, a 928 amino acid FarB, and a 301 amino acid FarC (Figure S3). Because FarC lacked the canonical C6 binding motif of FarA and FarC, we questioned its functionality but nevertheless created deletion and overexpression strains of *farC* along with those of *farA* and *farB*.

Deletion mutants were created by replacing the *farA*, *farB*, and *farC* coding regions with *A*. fumigatus *pyrG*. For overexpression strains, *farA*, *farB*, and *farC* were overexpressed at their native loci using the constitutively active *A. nidulans gpdA* promoter, *gpdA(p)*, with the *A. parasiticus pyrG* marker gene for selection (Figure S4). Transformants were confirmed by PCR (data not shown), Southern blots, and in the case of overexpression mutants, semiquantitative RT-PCR (Figure S5).

The confirmed mutants were evaluated for marker gene effects by growing the strains on media with and without UU (Palmer *et al.* 2010). The wild type and all mutants, except for ∆*farB* and ∆*farC*, were unaffected by the addition of UU. ∆*farB* and ∆*farC* were somewhat restricted in radial growth on minimal medium, but this was partially remediated by the addition of UU (Figure S6), suggestive of a marker gene effect. Therefore, all subsequent *in vitro* experiments were carried out with UU supplemented in the media for all strains. UU supplementation was not required for growth on maize kernels, as previous studies have shown that *A. flavus pyrG* mutants are equally pathogenic as wild type on maize kernels (Amaike *et al.* 2013).

### Expression of far genes in response to fatty acids

**Figure 2:**
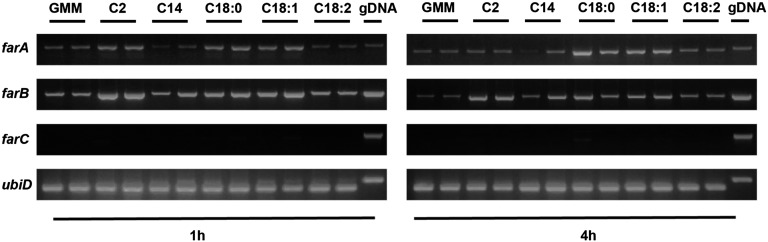
Expression of *far* genes on different carbon sources. Strains were grown for 24 hr in liquid GMM (NH_4_^+^) + UU, then transferred to fresh containing minimal medium (UU + 1% tergitol + 70.6 mM ammonium chloride) with 1% glucose (GMM), 50 mM acetic acid (C2), 2.5 mM myristic acid (C14), 2.5 mM stearic acid (C18:0), 2.5 mM oleic acid (C18:1). and 2.5 mM linoleic acid (C18:2). After 1 and 4 hr, mycelia were collected, respectively. RNA was extracted and converted to cDNA, and semiquantitative RT-PCR was performed. Expression of housekeeping gene *ubiD*, which encodes ubiquitin, was included as a control. cDNA, complementary DNA; gDNA, genomic DNA; GMM, glucose minimal medium; NH_4_^+^, ammonium; RT-PCR, reverse transcription polymerase chain reaction; UU, 5 mM uridine and 5 mM uracil.

To determine if *far* genes were responsive to fatty acid treatments, as has been seen with other fungi, wild-type *A. flavus* was grown in 1% GMM for 24 hr and transferred to fresh minimal medium containing a representative short- (acetic acid, C2), medium- (myristic acid, C14), saturated long-chain (stearic acid, C18:0), or unsaturated long-chain (oleic acid, C18:1; linoleic acid, C18:2) fatty acid. Therefore, the expression levels observed comprised the combined effect of glucose derepression and the differential response to the individual fatty acids. After 1 and 4 hr, the mycelia were collected. RNA was extracted, and semiquantitative RT-PCR was carried out to evaluate expression of *farA*, *farB*, and *farC. farA* and *farB* were differentially expressed depending on the fatty acid, with both showing higher expression with acetic acid (C2), stearic acid (C18:0), and oleic acid (C18:1) supplementation after 1 hr. The same trends were observed after 4 hr, except for *farA*, which showed only a transient response at 1 hr to acetic acid (C2). *farC* was not expressed in any conditions examined, in line with its likely nonfunctionality (Figure 2).

### Loss of farA and farB alters growth on fatty acids

To evaluate the roles of *farA*, *farB*, and *farC* in the metabolism of fatty acids of various chain lengths, the mutant strains were grown on minimal media containing short- (C2–C6), medium- (C12–C14), or long- (C16–C22) chain carboxylic acids as sole carbon sources. In addition to these media, strains were also grown on media containing Tween 20, Tween 80, or corn oil. Tween 20 and Tween 80 are surfactants with lauric acid (C12) and oleic acid (C18:1) ester-linked moieties, respectively. Corn oil is mainly composed of linoleic (C18:2), oleic (C18:1), and palmitic (C16:0) fatty acids as triglycerides, diglycerides, and free fatty acids (Strocchi 1982). We found that *farC* mutants had no impact on growth in any conditions and present data from this mutant in supplemental data (Figure S12).

**Figure 3:**
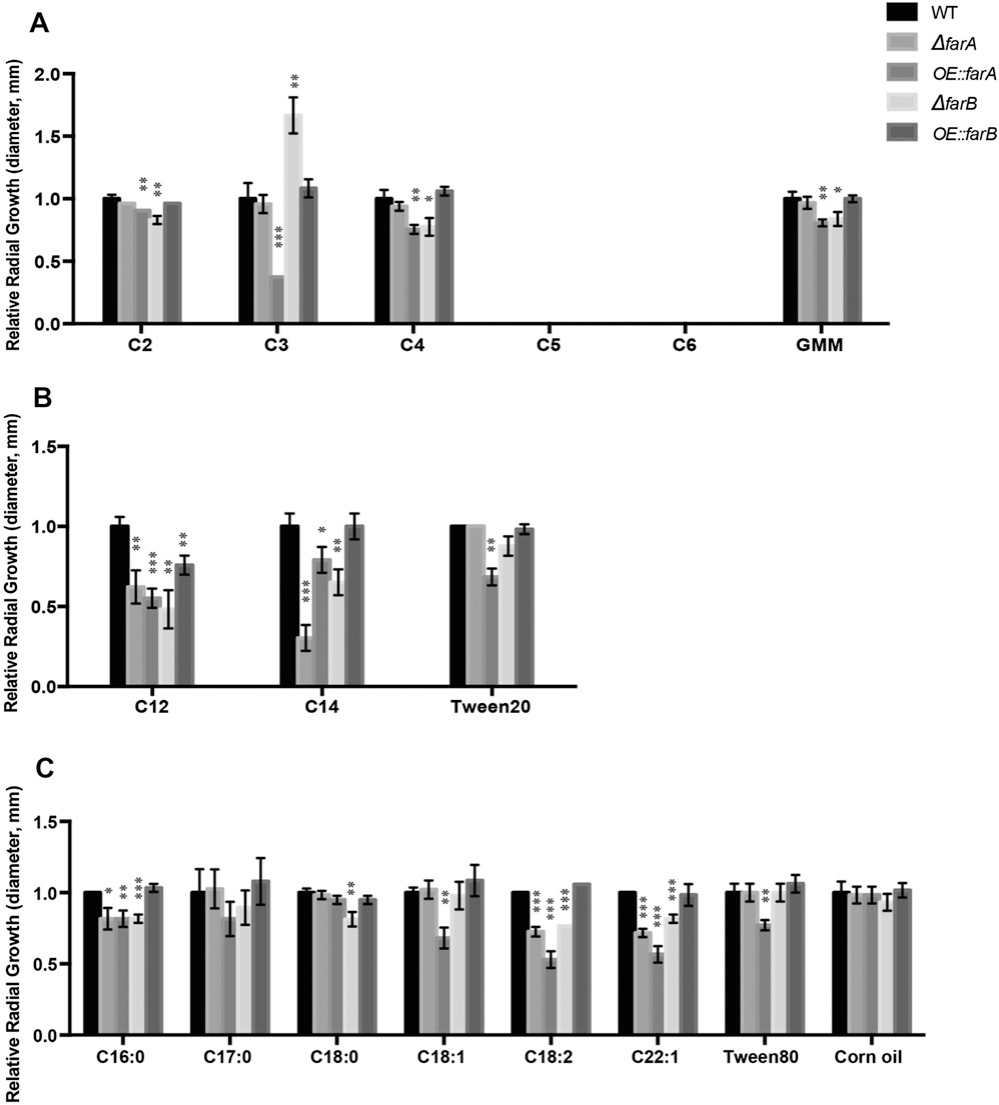
Growth of *farA* and *farB* mutants on carbon sources of various chain lengths. Strains were point-inoculated onto plates containing media with short-chain (A), medium-chain (B), and long-chain (C) carbon sources. After 3 d, radial growth measurements were taken. For all graphs, wild-type (WT) data were normalized to be 1. The length of the carbon chain is indicated on the *x*-axis, and the shades of the bars correspond to the legend on the right. Asterisks indicate statistical significance compared to the wild type for each condition as determined by a two-tailed Student’s *t*-test with * *P* < 0.05, ** *P* < 0.01, and *** *P* < 0.001. Bars lacking error bars indicate that identical values were observed in each growth test.

Depending on the media, both deletion and overexpression *farA* and *farB* mutants showed significant differences from the wild type. The strain showing the most difference from the wild type was the *OE*::*farA* mutant, which showed significant inhibition of growth on all fatty acids except margaric acid (C17:0) and stearic acid (C18:0) (Figure 3 and Figure S7). In contrast to its wild-type-like growth on stearic acid (C18:0), the *OE*::*farA* mutant was considerably impaired in growth on unsaturated C18 fatty acids with increasing restriction on the polyunsaturated linoleic acid (C18:2). The most striking phenotype of the ∆*farA* mutant was its greatly impaired ability to grow on medium-chain fatty acids [lauric acid (C12) and myristic acid (C14)]. The ∆*farB* mutant also exhibited reduced growth on medium-chain fatty acids and, to a lesser extent, on some short- and long-chain fatty acids (Figure 3). One unique observation was the increased radial growth of ∆*farB* on propionic acid (C3) media. This phenotype was coupled with sparse mycelial growth so that, despite larger radial growth, the strain appeared sicker than the wild type. The sicker growth phenotype was still observed when glucose was added to propionic acid medium (Figure S8). The *OE*::*farB* mutant grew similarly to the wild type on all fatty acids but lauric acid (C12).

No strains could grow on valeric acid (C5) and hexanoic acid (C6) as sole carbon sources. The strains were also unable to grow on GMM amended with valeric acid (C5) or hexanoic acid (C6) (data not shown). This suggests that valeric acid (C5) and hexanoic acid (C6) were toxic to *A. flavus*, at least as conducted in this study.

### Transcriptional profile of fatty acid metabolism genes regulated by FarA

Growth tests (Figure 3) suggested an important role for FarA in fatty acid metabolism. To examine the impact of FarA on the expression of genes involved in fatty acid metabolic pathways, *farA* mutants and the wild type were grown on both liquid and solid media treated with different fatty acids, as described in *Materials and Methods*. Semiquantitative RT-PCR was carried out to evaluate expression levels of genes involved in catabolic pathways including β-oxidation (*e.g.*, mitochondrial enoyl-CoA hydratase *echA*, mitochondrial acyl-CoA dehydrogenase *scdA*, and peroxisomal β-oxidation multifunctional enzyme *foxA*), peroxisomal proliferation (*e.g.*, peroxin *pexK*), fatty acid transport (*e.g.*, acetyl-carnitine transferase *acuJ*), acetate metabolism (*e.g.*, acetyl-CoA synthetase *facA*), and unsaturated fatty acid metabolism (*e.g.*, putative enoyl-CoA isomerase *eci*A and 2,4 dienoyl-CoA reductase *dcrA*). Anabolic genes assessed included *fasA* (*e.g.*, encoding the putative fatty acid synthase α-subunit), *sdeA* and *sdeB* (encoding the putative ∆-9-stearic acid desaturases), and *odeA* (encoding the putative oleate ∆-12 desaturase). The presence of putative Far binding sites (CCTCGG motif) (Hynes *et al.* 2006) in the 5′ region of the promoters of these genes is presented in Table 1.

**Table 1:** Presence of the Far CCTCGG binding motif in the 5′ region of *A. flavus* genes assessed on this study

Gene (Accession Number)	Gene Annotation	Position Relative to Start Codon
*farA* (AFL2G_05109 )	Zn2-Cys6 transcription factors	No motif found
*farB* (AFLA_012010 )	Zn2-Cys6 transcription factors	−916
*pexK* (AFLA_036410 )	Peroxisomal biogenesis factor, putative	−334
*echA* (AFLA_043610 )	Mitochondrial enoyl-CoA hydratase	−166
*scdA* (AFLA_084820 )	Mitochondrial acyl-CoA dehydrogenase	−158
*foxA* (AFLA_041590 )	Peroxisomal multifunctional β-oxidation protein (MFP), putative	−924
*acuJ* (AFLA_135240 )	Acetyl-carnitine transferase	−160, −361
*eciA* (AFLA_055840 )	Peroxisomal D3,D2-enoyl-CoA isomerase, putative	−311
*derA* (AFLA_061230 )	2,4 dienoyl-CoA reductase, putative	−179, −867
*facA* (AFLA_027070 )	Acetyl-CoA synthetase	No motif found
*aflR* (AFLA_139360 )	GAL4-like Zn2Cys6 transcription factor	−199
*aflD*(AFLA_139390 )	Reductase	−609
*fasA* (AFLA_089170 )	Fatty acid synthase α-subunit, putative	No motif found
*sdeA* (AFLA_076880 )	∆-9-stearic acid desaturase, putative	−35, −160, −448
*sdeB* (AFLA_004460 )	∆-9-stearic acid desaturase, putative	−422
*odeA* (AFLA_066330 )	Oleate ∆-12 desaturase, putative	−870

Positions of CCTCGG or the complement CCGAGG were obtained by scanning 1 kb upstream of the proposed start codon. Genes were from http://www.ncbi.nlm.nih.gov/ and http://www.aspgd.org.

**Figure 4:**
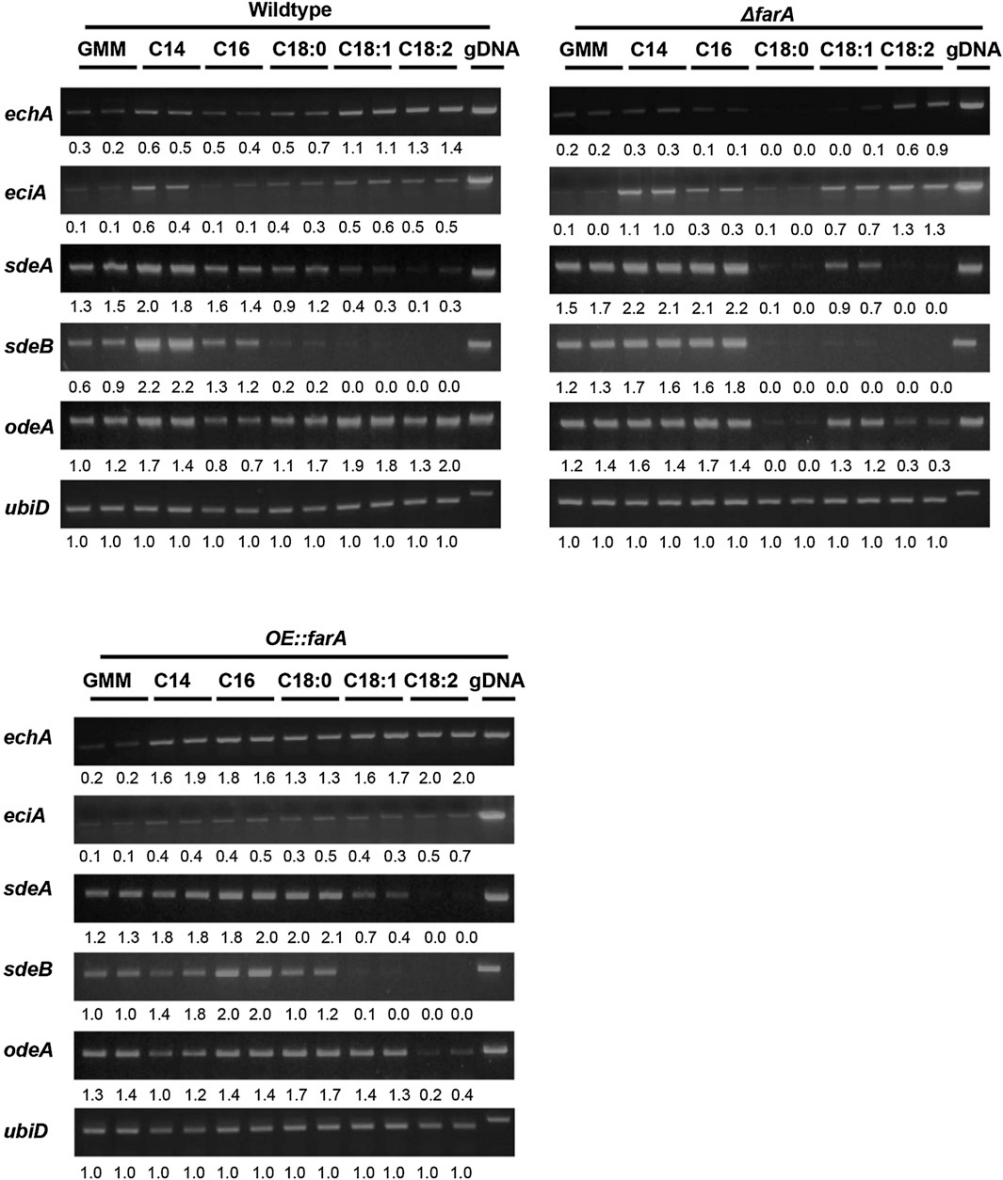
Expression of genes in *farA* mutants under different carbon sources. Strains were grown for 24 hr in liquid GMM (NH_4_^+^) + UU, then transferred to fresh containing minimal medium (UU + 1% tergitol + 70.6 mM ammonium chloride) with 1% glucose (GMM), 2.5 mM myristic acid (C14), 2.5 mM palmitic acid (C16), 2.5 mM stearic acid (C18:0), 2.5 mM oleic acid (C18:1), and 2.5 mM linoleic acid (C18:2). After 4 hr, mycelia were collected. RNA was extracted and converted to cDNA, and semiquantitative RT-PCR was performed. Expression of housekeeping gene *ubiD*, which encodes ubiquitin, was included as a control. Numbers below the bands indicate the quantified intensity of the band normalized to *ubiD* expression for each sample. cDNA, complementary DNA; gDNA, genomic DNA; GMM, glucose minimal medium; NH_4_^+^, ammonium; RT-PCR, reverse transcription polymerase chain reaction; UU, 5 mM uridine and 5 mM uracil.

Notably, in liquid culture, *farA* loss and overexpression had opposite impacts on the expression of the mitochondrial β-oxidation gene *echA* in most treatments, with *echA* expression diminished in the deletion strain but enhanced in the overexpression strain (Figure 4). The *OE*::*farA* strain showed reduced expression of the putative enoyl-CoA isomerase *eciA*, which could partly explain the reduced ability of this strain to grow on long-chain unsaturated fatty acids. Expression of most genes was not impacted by either *farA* loss or overexpression (Figure S9). In solid culture, the same expression pattern was also observed but to a lesser degree (Figure S11).

Anabolic genes were regulated by *farA* in a fatty acid-dependent manner. When grown on the unsaturated fatty acid stearic acid (18:0), *farA* loss led to decreased expression of all three desaturase genes, *sdeA*, *sdeB*, and *odeA* (Figure 4). Overexpression of FarA increased *sdeB* expression. In solid culture, the same expression pattern was observed in both C18:0 and C18:1 treatments (Figure S11).

### Transcriptional profile of fatty acid metabolism genes regulated by FarB

**Figure 5:**
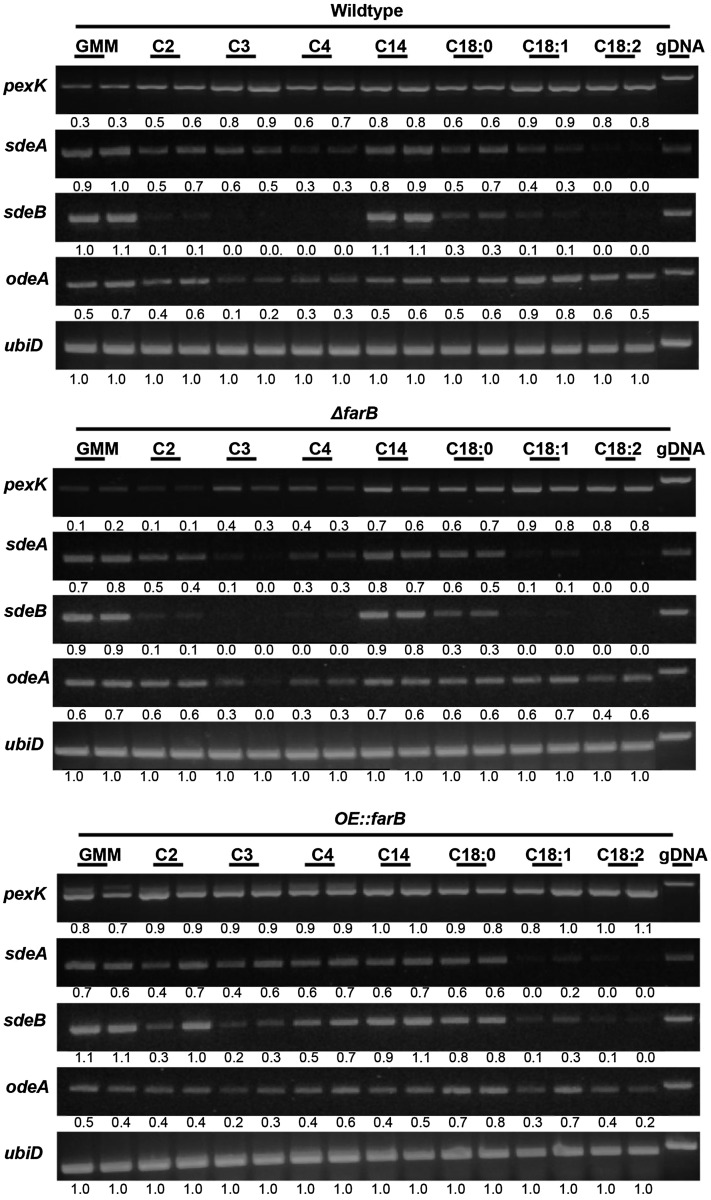
Expression of genes on *farB* mutants under different carbon sources. Strains were grown for 24 hr in liquid GMM (NH_4_^+^) + UU, then transferred to fresh containing minimal medium (UU + 1% tergitol + 70.6 mM ammonium chloride) with 1% glucose (GMM), 50 mM acetic acid (C2) and propionic acid (C3), 10 mM sodium butyrate (C4), 2.5 mM myristic acid (C14), 2.5 mM stearic acid (C18:0), 2.5 mM oleic acid (C18:1), and 2.5 mM linoleic acid (C18:2). After 4 hr, mycelia were collected. RNA was extracted and converted to cDNA, and semiquantitative RT-PCR was performed. Expression of housekeeping gene *ubiD*, which encodes ubiquitin, was included as a control. Numbers below the bands indicate the quantified intensity of the band normalized to *ubiD* expression for each sample. cDNA, complementary DNA; gDNA, genomic DNA; GMM, glucose minimal medium; NH_4_^+^, ammonium; RT-PCR, reverse transcription polymerase chain reaction; UU, 5 mM uridine and 5 mM uracil.

The growth test also suggested a possible broad role for FarB in fatty acid metabolism, although the growth defects with the ∆*farB* strain across almost all media tested, including GMM (Figure 3), complicated the analysis of this strain. To gain more insight into the fatty acid metabolism processes regulated by this transcription factor, we examined gene expression in both deletion and overexpression *farB* mutants in an expanded range of fatty acids in liquid culture including acetic acid (C2), propionic acid (C3), sodium butyrate (C4), myristic acid (C14), stearic acid (C18:0), oleic acid (C18:1), and linoleic acid (C18:2). We also grew the strains in solid culture in the same manner as *farA* mutants. Semiquantitative RT-PCR was carried out to evaluate expression levels of genes involved in β-oxidation (*echA*, *scdA*, and *foxA*), peroxisomal proliferation (*pexK*), fatty acid transport (*acuJ*), and acetate metabolism (*facA*) under these conditions. Gene expression compared to the wild type was not impacted to any great extent in either liquid or solid grown cultures (Figure S10 and data not shown), aside from slightly enhanced expression of *pexK* in the *OE*::*farB* strain and reduced induction of *pexK* by short-chain fatty acids in ∆*farB* in liquid culture (Figure 5).

FarB mutants had little impact on anabolic gene expression, with the exception of enhanced *sdeB* expression on short-chain fatty acids in both liquid and solid culture. (Figure 5, Figure S10, and Figure S11).

### In vitro aflatoxin production and differential expression of genes in far mutants

**Figure 6:**
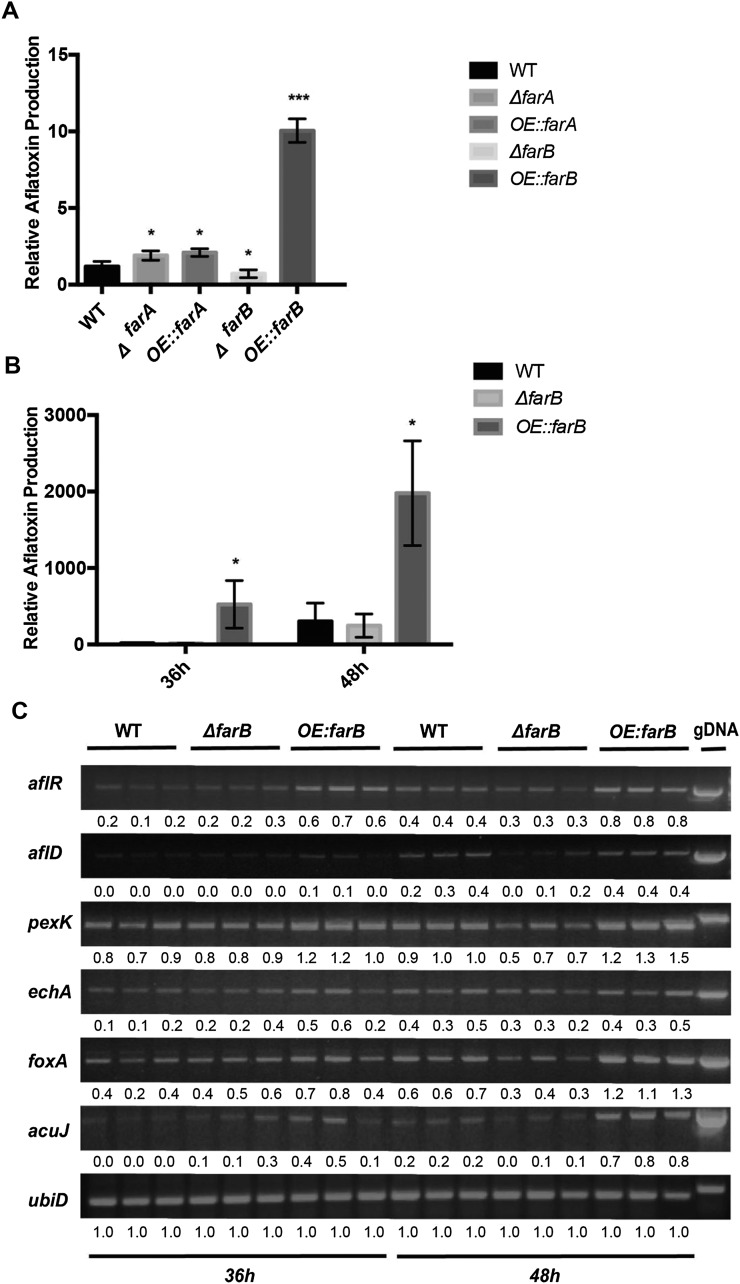
*In vitro* aflatoxin production by *farA* and *farB* mutants and expression of genes on *farB* mutants under liquid YES medium. (A) Strains were grown on AF-inducing solid YES media for 3 d, after which AF was extracted and quantified by HPLC. (B) Strains were grown in 50 ml YES + UU in 125 ml flasks. Three replicates per strain were shaken in the dark at 29° and 250 rpm. AF was extracted from flasks after 36 and 48 hr, respectively, and quantified by HPLC. The shades of the bars correspond to the legend on the right. Asterisks represent statistical significance compared to the wild type as determined by a two-tailed Student’s *t*-test with * *P* < 0.05, ** *P* < 0.01, and *** *P* < 0.001. (C) The mycelia from the same flasks as for AF extraction were collected. RNA was extracted and converted to cDNA, and semiquantitative RT-PCR was performed. Expression of housekeeping gene *ubiD*, which encodes ubiquitin, was included as a control. Numbers below the bands indicate the quantified intensity of the band normalized to *ubiD* expression for each sample. AF, aflatoxin; cDNA, complementary DNA; gDNA, genomic DNA; HPLC, high performance liquid chromatography; RT-PCR, reverse transcription polymerase chain reaction; UU, 5 mM uridine and 5 mM uracil; WT, wild type; YES, 2% yeast extract and 6% sucrose, pH 5.8.

The *far* mutants were also tested for their ability to produce AF *in vitro*. The mutants were grown in solid YES medium, which supports AF production. High Performance Liquid Chromatography (HPLC) showed that deletion of *farB* yielded a reduction of AF compared to the wild type. Both deletion and overexpression of *farA* resulted in slight increases in AF. However, the most striking increase was with *OE*::*farB*, which made approximately fivefold more AF than the wild type (Figure 6A). AF production of *farB* mutants was also tested in liquid YES medium. Here, the *OE*::*farB* strain produced an even higher fold increase in AF (Figure 6B). The *farC* mutants did not show a differential AF production compared to the wild type (Figure S12). AF biosynthesis on AF-repressive YEP medium was below the limit of detection for all strains (data not shown).

To obtain some understanding of why AF was increased to such high levels in *OE*::*farB*, we assessed expression of the AF regulatory transcriptional activator gene *aflR* (Woloshuk *et al.* 1994) and a key structural gene encoding an enzyme that catalyzes the conversion of norsolorinic acid to averantin in the AF biosynthetic pathway *aflD* (formerly called nor-1, Trail *et al.* 1994), as well as genes that are involved in processes leading to increases in acyl-CoA pools, the precursors for aflatoxin synthesis. These latter genes include the β-oxidation gene *foxA*, the peroxisomal proliferation gene *pexK*, and the acetyl-carnitine transferase *acuJ*. Using the same conditions as shown in Figure 6B, we found that loss of *farB* resulted in decreases, while overexpression of *farB* resulted in increases, in expression of nearly all of the genes tested compared to the wild type after 48 hr shaking growth conditions (Figure 6C). Thus, it appeared that FarB positively regulates not only *aflD* and *aflR*, but also genes involved in generating precursor pools (*e.g.*, acyl CoA units) for the polyketide AF.

Although all of these genes contained putative FarB binding motifs in their promoters (Table 1), because they were not increased in expression in the *OE*::*farB* mutant in fatty acid treatments (Figure 5, Figure S10, and Figure S11), we speculated that other pathways could be involved in their regulation in YES medium. Many genes are repressed by the carbon catabolite repressor CreA in high sucrose medium, thus, we looked for the presence of putative CreA consensus binding sites (Cubero and Scazzocchio 1994) in the 5′ region of the promoters of these genes (Table S4). We found putative CreA binding sites in *pexK*, *foxA*, and *acuJ*, the three precursor pool related genes most clearly regulated by FarB. Possibly, FarB could override any repressive role of CreA in regulation of these genes.

### Colonization on maize kernels

**Figure 7:**
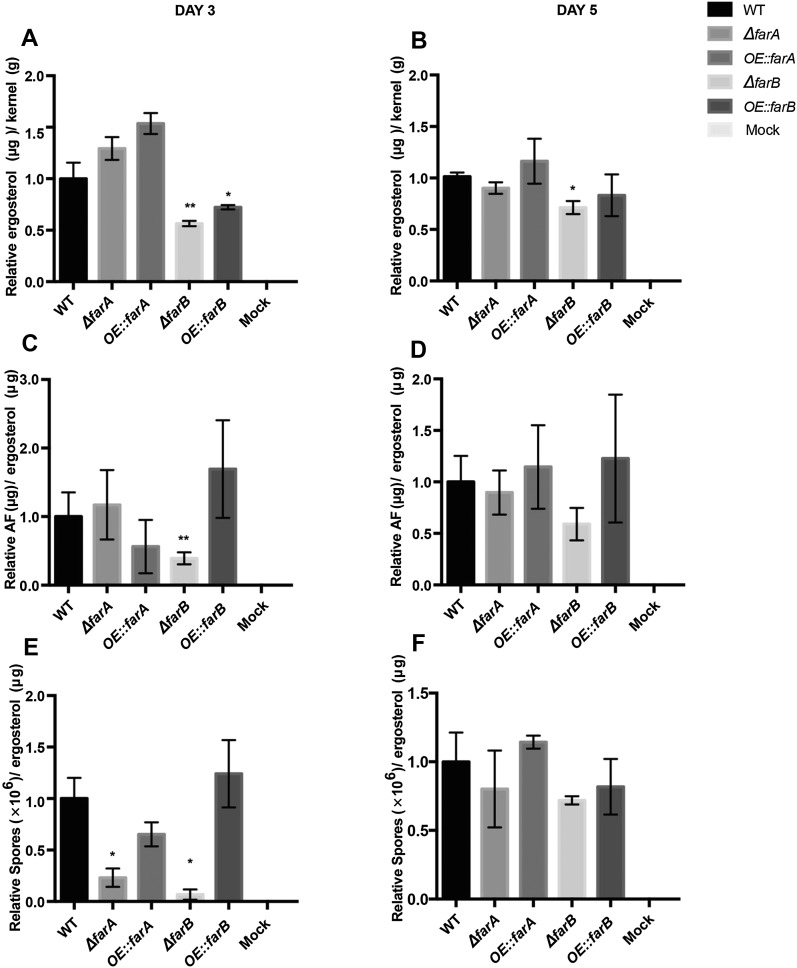
The effects of *farA* and *farB* on virulence on maize kernels. Maize kernels were infected with *far* mutant strains. Ergosterol was extracted after 2 (A) and 3 d (B), respectively, and quantified by HPLC. Aflatoxin was also extracted at 2 (C) and 3 d (D), respectively, and quantified by HPLC. Spores were washed from the kernels after 2 (E) and 3 d (F), respectively, and counted. For all graphs, wild-type data were normalized to be 1. The shades of the bars correspond to the legend on the right. Asterisks indicate statistical significance compared to wild type as determined by a two-tailed Student’s *t*-test with * *P* < 0.05, ** *P* < 0.01, and *** *P* < 0.001. HPLC, high performance liquid chromatography; WT, wild type.

The *A. flavus far* mutants were also tested for their ability to infect maize kernels. Growth was assessed by quantifying the fungus-specific sterol ergosterol. AF production and sporulation on the kernels were also measured and normalized to fungal burden (*i.e.*, ergosterol content). After 3 d, ∆*farB* was impaired in growth (Figure 7A), AF production (Figure 7C), and sporulation (Figure 7E) on maize kernels. Reduced growth of this mutant was still observed at 5 d postinoculation (DPI) (Figure 7B). *OE*::*farB* was also impaired in growth at 3 DPI (Figure 7A). Unlike the significant increase of AF in the *OE*::*farB* strain on YES medium (Figure 6), this strain did not produce more AF corn kernels at the time points tested (Figure 7, C and D). ∆*farA* and ∆*farC* also showed decreases in sporulation at 3 DPI (Figure 7E and Figure S13). *farA* and *farC* had no observable impacts on fungal ingress or AF production (Figure 7E and Figure S13). There were also no significant differences in sporulation by any of the mutants at 5 DPI (Figure 7F and Figure S13).

## Discussion

The ability of *A. flavus* to metabolize lipids is a critical factor for its success as a seed pathogen (Amaike and Keller 2009; Scarpari *et al.* 2014). Here, we examined homologs of previously identified Zn2-Cys6 transcription factors (Far proteins) that regulate fatty acid metabolism in other fungi for their roles in *A. flavus* growth, AF production, and virulence on maize kernels. An investigation of the *A. flavus* genome suggested the presence of three, not two, *far* genes (Figure 1). However, FarC lacks the critical Zn2-Cys6 domain for binding to DNA, is not expressed under the conditions examined in this study, nor does its deletion or overexpression have any notable impact on fungal growth. Therefore, we suggest that FarC may be a remnant of a duplication of FarA with no function in this species. *A. flavus* and *A. oryzae* are known to contain extra copies of many genes compared to other *Aspergillus* spp., although the functionality of these ‘extra’ copies is rarely studied (Khaldi and Wolfe 2008; Hammond *et al.* 2008).

Despite being conserved across many species of fungi, and always involved in the regulation of fatty acid metabolism, the exact roles of FarA and FarB on these regulatory processes vary depending on the fungus and growth conditions. For example, in *A. nidulans*, loss of *farA* resulted in growth defects on all lengths of fatty acids tested (Hynes *et al.* 2006). In *A. oryzae*, however, a ∆*farA* strain grew like the wild type except for a slight defect on long-chain fatty acids (Garrido *et al.* 2012). In *M. oryzae*, the disruption of the *farB* ortholog, *far2*, resulted in inability to utilize acetate as a sole carbon source (Bin Yusof *et al.* 2014), while *A. nidulans* can still utilize acetate without *farB* (Hynes *et al.* 2006). Our results with *A. flavus* showed a similar phenotype to the ∆*farA* mutant in *A. nidulans*, where *A. flavus* ∆*farA* was also defective in growth on most fatty acids compared to the wild type (Figure 3); this was true for *farB* as well, although to a lesser degree. Overexpression of *farA* showed growth defects on most media, not limited to fatty acid-amended media, which may indicate a complicated mechanism of action of this transcription factor beyond lipid metabolic pathways. Supporting their requirement for normal growth on fatty acid supplemented media, we found that *farA* and *farB* were differentially expressed dependent on fatty acid treatment (Figure 2). In contrast, *farC* was not expressed in any condition. This lack of expression was consistent with a lack of phenotype of *farC* mutants as assayed in our hands.

The *farA* mutants showed particularly strong phenotypes on medium-chain and unsaturated fatty acid metabolism where loss of *farA* resulted in very poor growth on lauric acid (C12) and myristic acid (C14), and the *OE*::*farA* strain, while growing well on stearic acid (C18:0), was severely impaired on growth on saturated C18 species. To gain insight into these phenotypes, we examined the expression of genes involved in fatty acid metabolism by medium- and long-chain fatty acids in the *farA* mutants. Our results suggest that FarA may have a regulatory role in mitochondrial β-oxidation and monounsaturated fatty acid biosynthesis, as supported by the reduced expression of genes important in these processes (Figure 4). The mitochondrial enoyl-CoA hydratase *echA* is required for mitochondrial β-oxidation (Maggio-Hall and Keller 2004) and is considerably downregulated in the ∆*farA* mutant, which may explain some of the phenotype of this strain. FarA also appears to have a possible negative role in expression of the putative enoyl-CoA isomerase, as *eciA* is downregulated in the OE::*farA* strain, which grows poorly on unsaturated C:18 species.

∆*farB* also showed a growth defect on most of the fatty acid media tested while *OE*::*farB* grew similarly to the wild type. Our analysis indicated an impact of FarB on several lipid metabolism genes that was dependent on media. When the *farB* mutants were grown on fatty acid supplemented media, there was little difference in catabolic gene expression compared to the wild type, at least at the time point chosen (Figure 5), whereas when these same mutants were grown in YES medium, FarB was found to positively regulate several genes involved in β-oxidation processes (Figure 6). However, one gene positively regulated by FarB in both media was *pexK*, which is involved in peroxisomal proliferation. This result is reminiscent of studies of regulation by Far transcription factors on peroxisomal biogenesis in *A. nidulans* (Hynes *et al.* 2006; Hynes *et al.* 2008).

In addition to Far regulation of fatty acid catabolic pathways, we also examined the *far* mutants for impacts on anabolic pathways. As far as we know, such studies have not previously been conducted on *far* mutants of other fungi. The stearic acid desaturases SdeA and SdeB, and oleate desaturase OdeA, are required for synthesis of the polyunsaturated fatty acids linoleic acid and oleic acid, respectively. Although not studied in *A. flavus*, deletion of *ode*A in two isolates of the closely related species *A. parasiticus* resulted in mutants impaired in various parameters of colony growth as well as virulence on corn (Wilson *et al.* 2004a). Deletion of *sdeA* in *A. nidulans* greatly reduced its ability to grow and a double deletion of both *sdeA* and *sdeB* was lethal (Wilson *et al.* 2004b). We found that FarA positively regulated all three desaturase genes when grown on stearic acid (Figure 4 and Figure S11), although a change in *farA* mutant growth was not seen on this medium (Figure 3). Inexplicably, overexpression of *farB* increased *sdeB* expression on C2, C3, and C4 fatty acid treatments, but whether this increase is related to the unexpected phenotype of this strain on C3 is unknown.

An assessment of *far* transcription factors in secondary metabolism has not been discussed in general but can be implicated from work in *A. nidulans*. In *A. nidulans*, FarA and FarB regulate the expression of peroxisome biogenesis and β-oxidation genes (Hynes *et al.* 2006), and proliferation of peroxisomes is dependent on FarA (Hynes *et al.* 2008). Peroxisome proliferation and β-oxidation are correlated with production of AF and its precursor sterigmatocystin (ST), as β-oxidation catabolizes fatty acids to units of acetyl-CoA, the starting material for polyketide synthesis. The AF/ST precursor norsolorinic acid (NOR) accumulates in the peroxisomes of *A. flavus*, *A. nidulans*, and *A. parasiticus* (Maggio-Hall *et al.* 2005), and induction of peroxisomal β-oxidation and peroxisome proliferation, associated with increased expression of *pexK/pex11* and *foxA*, also increased AF production (Reverberi *et al.* 2012). Furthermore, exogenous oleic acid stimulates the production of ST, the penultimate precursor of AF, in *A. nidulans*, and this is dependent on both mitochondrial and peroxisomal β-oxidation pathways (Maggio-Hall *et al.* 2005). FarA also regulates suberin-degrading enzymes speculated to contribute to secondary metabolism (Martins *et al.* 2014). In *F. oxysporum* f. sp. *lycopersici*, the polyketide pigment bikaverin is induced by wheat germ oil, with its production lost in the absence of *ctf1* (Bravo-Ruiz *et al.* 2013).

Here, we find that FarB positively regulates AF production, at least on YES medium. This regulation appears to be both by positive regulation of genes in the AF biosynthetic pathway (*aflD* and *aflR*), as well as those genes involved in acyl-CoA genesis. *pexK*, *foxA*, and *acuJ* all encode proteins associated with peroxisomal and/or β-oxidation pathways that contribute to increasing acetyl-CoA pools, a known precursor of polyketides including AF. The presence of both FarB and CreA binding sites in the promoters of *pexK*, *foxA*, and *acuJ* may suggest an interaction of these two transcription factors in the regulation of these genes in YES medium. However, more extensive studies would be required to tease apart any interacting regulatory networks of these two pathways on gene expression.

We examined all mutants for their ability to colonize maize seed through measurement of the fungal sterol ergosterol, which has been found to be a reliable determination for fungal ingress (Christensen *et al.* 2012). Aflatoxin and spore numbers were assessed as a function of total ergosterol to present a view of toxin-producing and sporulation potential in the mutants. Both deletion and overexpression of *farB* showed a reduction in ergosterol production on seed at day three, with ∆*farB* still showing a reduction by day five, thus implicating FarB as playing a role in colonization of maize seed. Our assessment of growth on fatty acids and gene expression analysis could not provide a clear basis for why ∆*farB* would be less able to colonize seed than *farA* mutants; possibly, this is related to other parameters known to impact growth on seed such as reactive oxygen species (ROS) or nitrogen utilization (Amaike *et al.* 2013; Zhang *et al.* 2016). Although all deletion mutants were delayed in sporulation on maize kernels at 3 DPI, by 5 DPI there was no significant difference in sporulation, and there was little impact on aflatoxin synthesis during growth on seed.

Far regulation of fatty acid metabolism and/or aflatoxin synthesis may intersect with other known proteins involved in these processes. Microarray data indicates that LaeA is a negative regulator of *farB* (Georgianna *et al.* 2010), and the decreased/increased expression of *farB* in the ∆*laeA/OE*::*laeA* mutants, respectively, may contribute to the decreased/increased aflatoxin and virulence phenotypes of the *laeA* mutant strains (Amaike and Keller 2009; Kale *et al.* 2008). Oxylipins have been shown to play an important role in host–pathogen cross talk (Tsitsigiannis and Keller 2006; Brown *et al.* 2009). The regulatory effects of FarA and FarB on anabolic genes (Figure 4 and Figure 5) whose products are the substrates for oxylipin production suggest that Far proteins may network with oxylipin pathways as well.

Overall, the Far transcription factors appear to be conserved in their regulatory role in lipid metabolism, but their individual functions vary between fungi. This could contribute to the differences in virulence and host specificity seen in various fungi, as these transcription factors are vital for growing on particular sources of carbon. They also seem to provide an important link between primary and secondary metabolism, highlighting the need for deepening our understanding of the regulatory networks they control.

## Supplementary Material

Supplemental Material
