# Supplementary material for: Characterization of the Far Transcription Factor Family in Aspergillus flavus
Source: G3 (Bethesda). 2016 Aug 16;6(10):3269–81. doi: 10.1534/g3.116.032466 (PMC5068947; doi:10.1534/g3.116.032466)
Supplement: Supplemental Material [file supp_6_10_3269__index.html]

Characterization of the Far Transcription Factor Family in Aspergillus flavus — Supplemental Material 

# Characterization of the Far Transcription Factor Family in *Aspergillus flavus*

## Supplemental Material for Luo, *et al*, 2016

**Files in this Data Supplement:**

- Figure S1 - Region of colinearity of *farC*. (.pdf, 454 KB)
- Figure S10 - Expression of genes on *farB* mutants under different carbon sources. (.pdf, 215 KB)
- Figure S11 - Expression of genes on *farA* and *farB* mutants on solid culture supplemented by different fatty acids. (.pdf, 192 KB)
- Figure S12 - The effects of *farC* on fungal growth on carbon sources of various chain lengths, AF production. (.pdf, 219 KB)
- Figure S13 - The effects of *farC* on virulence on maize kernels. (.pdf, 262 KB)
- Table S1 - All strains used in this study and their genotypes. (.pdf, 126 KB)
- Table S2 - Oligonucleotide primers used for strain construction and confirmation. (.pdf, 148 KB)
- Table S3 - Semi-quantitative RT-PCR oligonucleotide primers and cycling conditions. (.pdf, 100 KB)
- Table S4 - Presence of putative CREA consensus binding sequence 5′-(C/G)YGGRG-3′ in the 5ï¿½ region of *A. flavus* genes assessed on this study. (.pdf, 186 KB)
- Figure S2 - Functional domains and multiple sequence alignment of Far proteins in *A. flavus.* (.pdf 283 KB)
- Figure S3 - *A. flavus* FarA (AFL2G\_05109) protein obtained from AspGD. (.pdf, 89 KB)
- Figure S4 - *farA, farB* and *farC* mutant constructs. (.pdf, 124 KB)
- Figure S5 - Strain confirmation. (.pdf, 292 KB)
- Figure S6 - *farA, farB* and *farC* mutant marker gene effects. (.pdf, 124 KB)
- Figure S7 - Growth of *far* mutants and the wildtype on fatty acids as sole carbon sources. (.pdf, 176 KB)
- Figure S8 - Growth test of *farB* mutants treated with propionic acid (C3). (.pdf, 120 KB)
- Figure S9 - Expression of genes on *farA* mutants under different carbon sources. (.pdf, 160 KB)
