# Supplementary material for: Characterization of the Far Transcription Factor Family in Aspergillus flavus
Source: G3 (Bethesda). 2016 Aug 16;6(10):3269–81. doi: 10.1534/g3.116.032466 (PMC5068947; doi:10.1534/g3.116.032466)
Supplement: Supplemental Material [file supp_g3.116.032466_FigureS1.pdf]

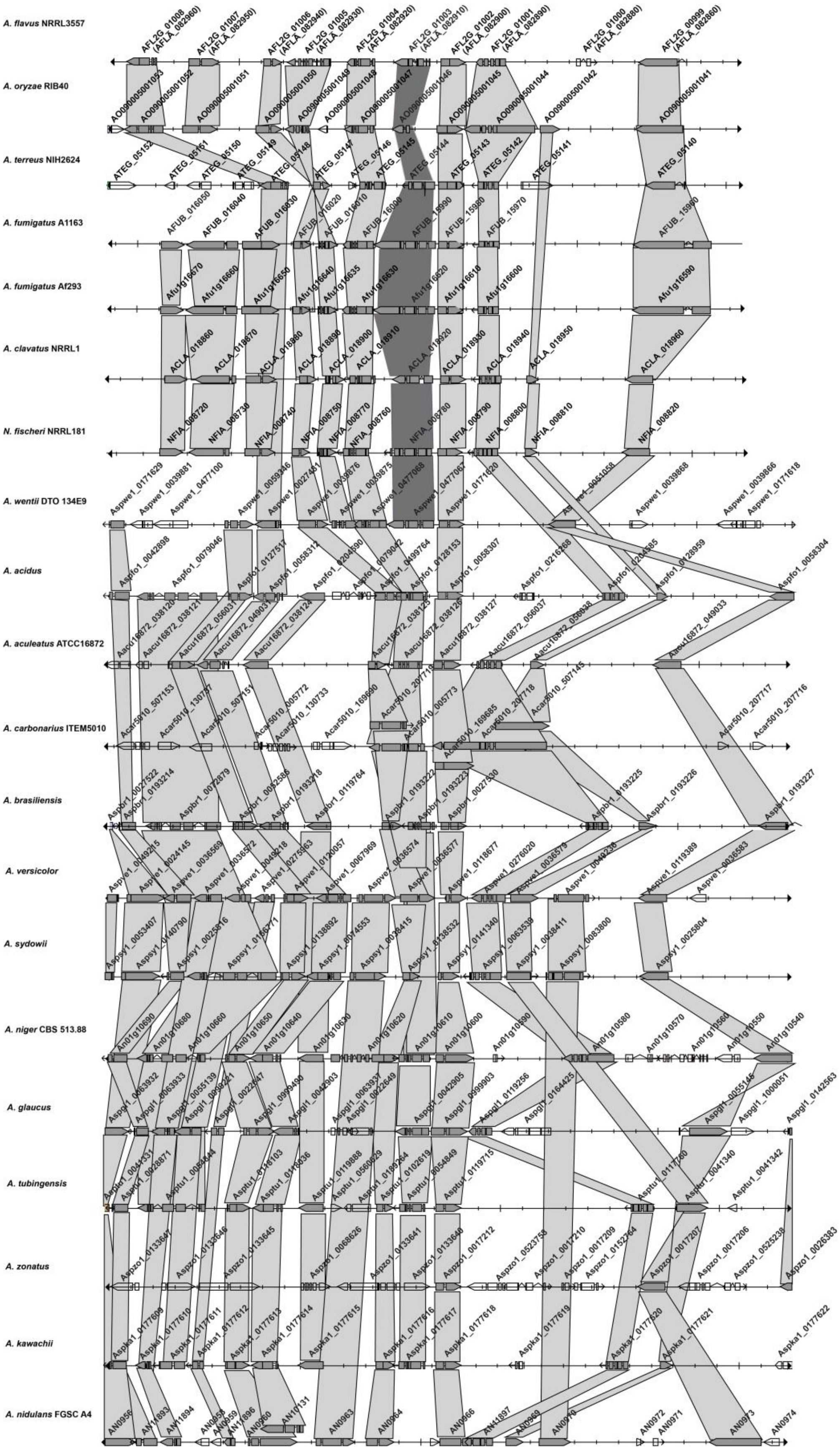
A. flavus NRRL3557
A. oryzae RIB40
A. terreus NIH2624
A. fumigatus A1163
A. fumigatus Af293
A. clavatus NRRL1
N. fischeri NRRL181
A. wentii DTO 134E9
A. acidus
A. aculeatus ATCC16872
A. carbonarius ITEM5010
A. brasiliensis
A. versicolor
A. sydowii
A. niger CBS 513.88
A. glaucus
A. tubingensis
A. zonatus
A. kawachii
A. nidulans FGSC A4

Figure S1 Region of colinearity of *farC*. The colinearity analysis was done from data obtained from AspGD website http://www.aspergillusgenome.org. *farC* homologues were identified from various *Aspergillus* spp. and *Neosartorya fischeri*. Vertical shading area indicates orthology. Darker shading area indicates orthology of *farC*. Arrow heads indicate direction of transcription.
