# Supplementary material for: Characterization of the Far Transcription Factor Family in Aspergillus flavus
Source: G3 (Bethesda). 2016 Aug 16;6(10):3269–81. doi: 10.1534/g3.116.032466 (PMC5068947; doi:10.1534/g3.116.032466)
Supplement: Supplemental Material [file supp_g3.116.032466_FigureS10.pdf]

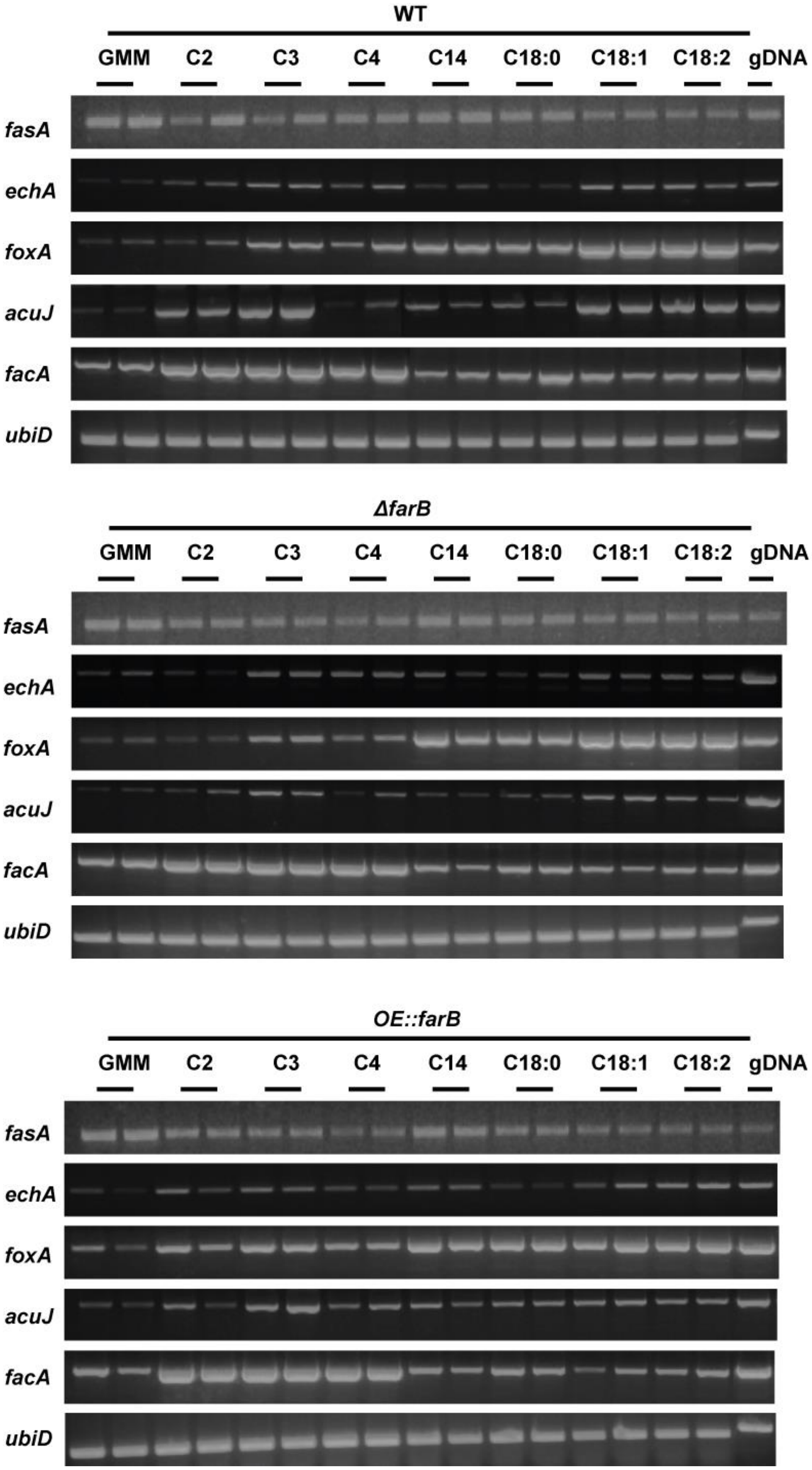
WT
GMM C2 C3 C4 C14 C18:0 C18:1 C18:2 gDNA
fasA
echA
foxA
acuJ
facA
ubiD
ΔfarB
GMM C2 C3 C4 C14 C18:0 C18:1 C18:2 gDNA
fasA
echA
foxA
acuJ
facA
ubiD
OE::farB
GMM C2 C3 C4 C14 C18:0 C18:1 C18:2 gDNA
fasA
echA
foxA
acuJ
facA
ubiD

Figure S10 Expression of genes on *farB* mutants under different carbon sources. Strains were grown for 24 hours in liquid GMM(NH_4^+)+UU, then transferred to fresh containing minimal medium (UU+1% tergitol+70.6 mM ammonium chloride) with 1% glucose, 50 mM acetic acid (C2) and propionic acid (C3), 10 mM sodium butyrate (C4), 2.5 mM myristic acid (C14), 2.5 mM stearic acid (C18:0), 2.5 mM oleic acid (C18:1) and 2.5 mM linoleic acid (C18:2). After four hours, mycelia were collected. RNA was extracted and converted to cDNA, and semi-quantitative RT-PCR was performed. Expression of housekeeping gene *ubiD,* which encodes ubiquitin, was included as a control.
