# Supplementary material for: Characterization of the Far Transcription Factor Family in Aspergillus flavus
Source: G3 (Bethesda). 2016 Aug 16;6(10):3269–81. doi: 10.1534/g3.116.032466 (PMC5068947; doi:10.1534/g3.116.032466)
Supplement: Supplemental Material [file supp_g3.116.032466_FigureS11.pdf]

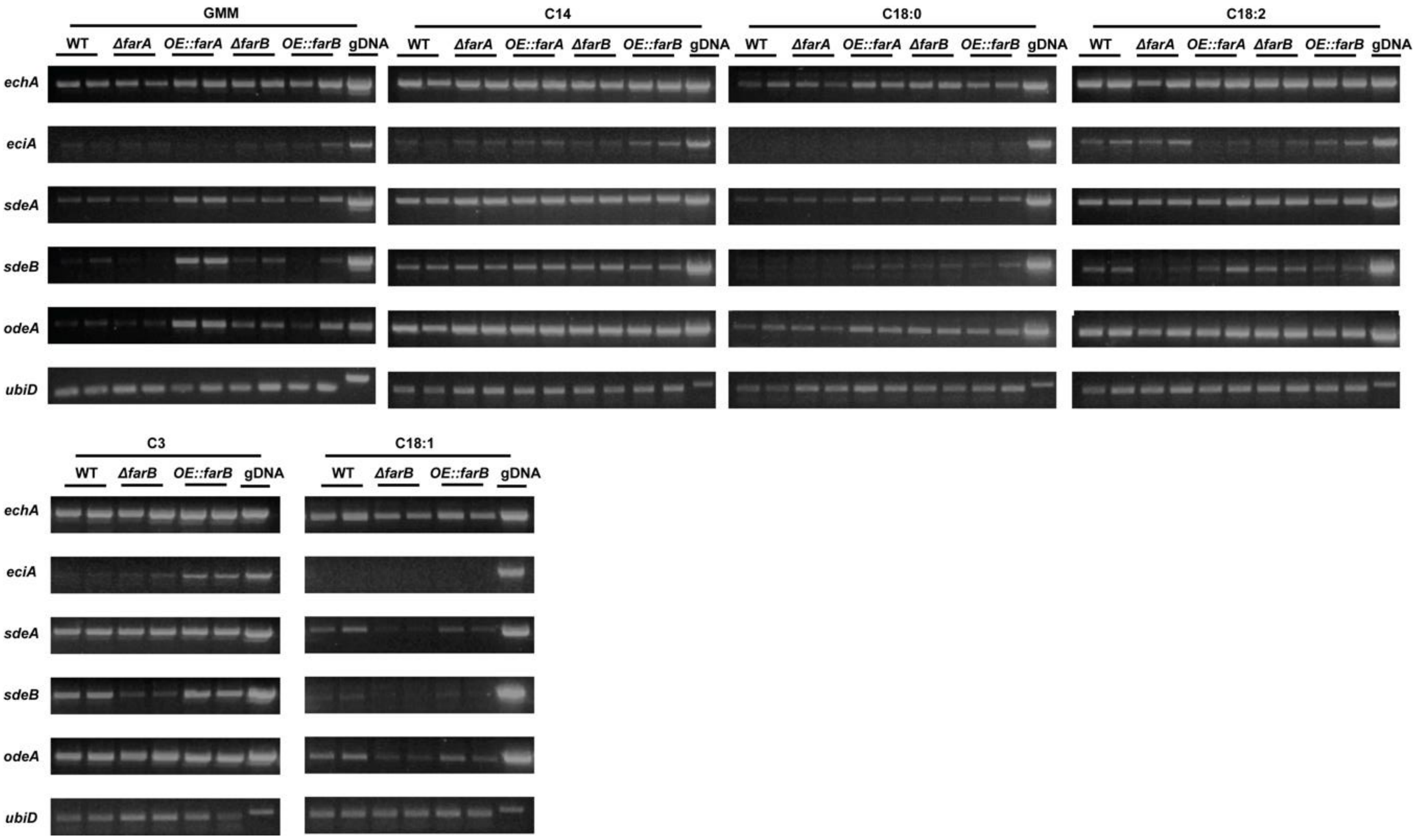
GMM
C14
C18:0
C18:2
WT
ΔfarA
OE::farA
ΔfarB
OE::farB
gDNA
echA
eciA
sdeA
sdeB
odeA
ubiD
C3
C18:1

Figure S11 Expression of genes on *farA* and *farB* mutants on solid culture supplemented by different fatty acids. The following carbon sources were added to minimal medium (UU+1% tergitol+70.6 mM ammonium chloride) with glucose (1%); propionic acid (C3) (50 mM); myristic (C14) (2.5mM); stearic (C18:0) (2.5mM); oleate (C18:1) (2.5mM) and linoleic (C18:2) (2.5mM); The surface of the medium was covered with cellophane sheets. After 3 days, the mycelium were collected. RNA was extracted and converted to cDNA, and semi-quantitative RT-PCR was performed. Expression of housekeeping gene *ubiD,* which encodes ubiquitin, was included as a control.
