# Supplementary material for: Characterization of the Far Transcription Factor Family in Aspergillus flavus
Source: G3 (Bethesda). 2016 Aug 16;6(10):3269–81. doi: 10.1534/g3.116.032466 (PMC5068947; doi:10.1534/g3.116.032466)
Supplement: Supplemental Material [file supp_g3.116.032466_FigureS12.pdf]

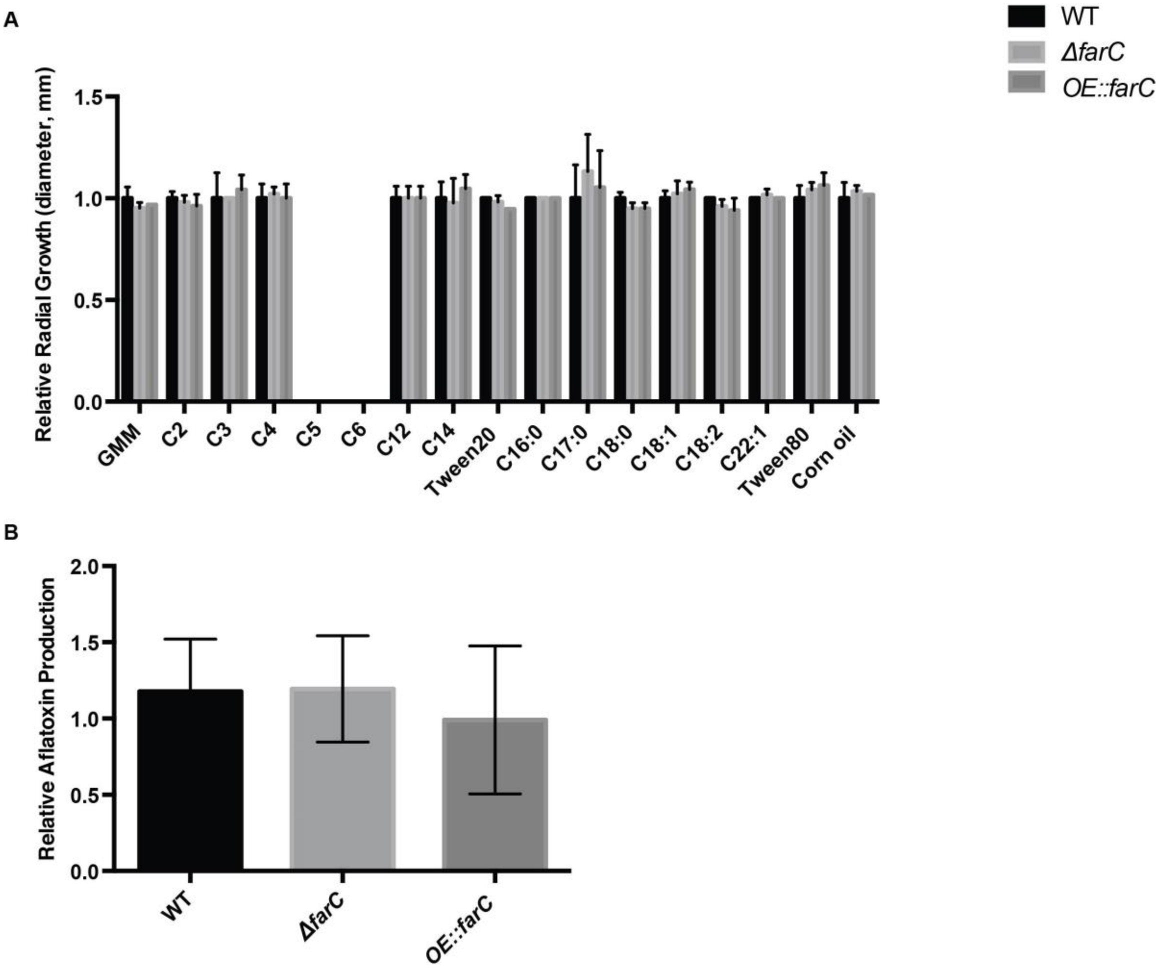

Figure S12 The effects of *farC* on fungal growth on carbon sources of various chain lengths, AF production. (A) *farC* mutants and wildtype strains were point-inoculated onto plates containing media with short-chain, medium-chain and long-chain carbon sources. After three days, radial growth measurements were taken. For all graphs, wildtype data was normalized to be 1. The length of the carbon chain is indicated on the x-axis, and the shades of the bars correspond to the legend on the right. Asterisks indicate statistical significance compared to the wildtype for each condition as determined by a two-tailed Student's T-test with $*p < 0.05$, $**p < 0.01$, and $***p < 0.001$. (B) *farC* mutants and wildtype strains were grown on AF-inducing solid YES media for three days, after which AF was extracted and quantified by HPLC.
