# Supplementary material for: Characterization of the Far Transcription Factor Family in Aspergillus flavus
Source: G3 (Bethesda). 2016 Aug 16;6(10):3269–81. doi: 10.1534/g3.116.032466 (PMC5068947; doi:10.1534/g3.116.032466)
Supplement: Supplemental Material [file supp_g3.116.032466_FigureS13.pdf]

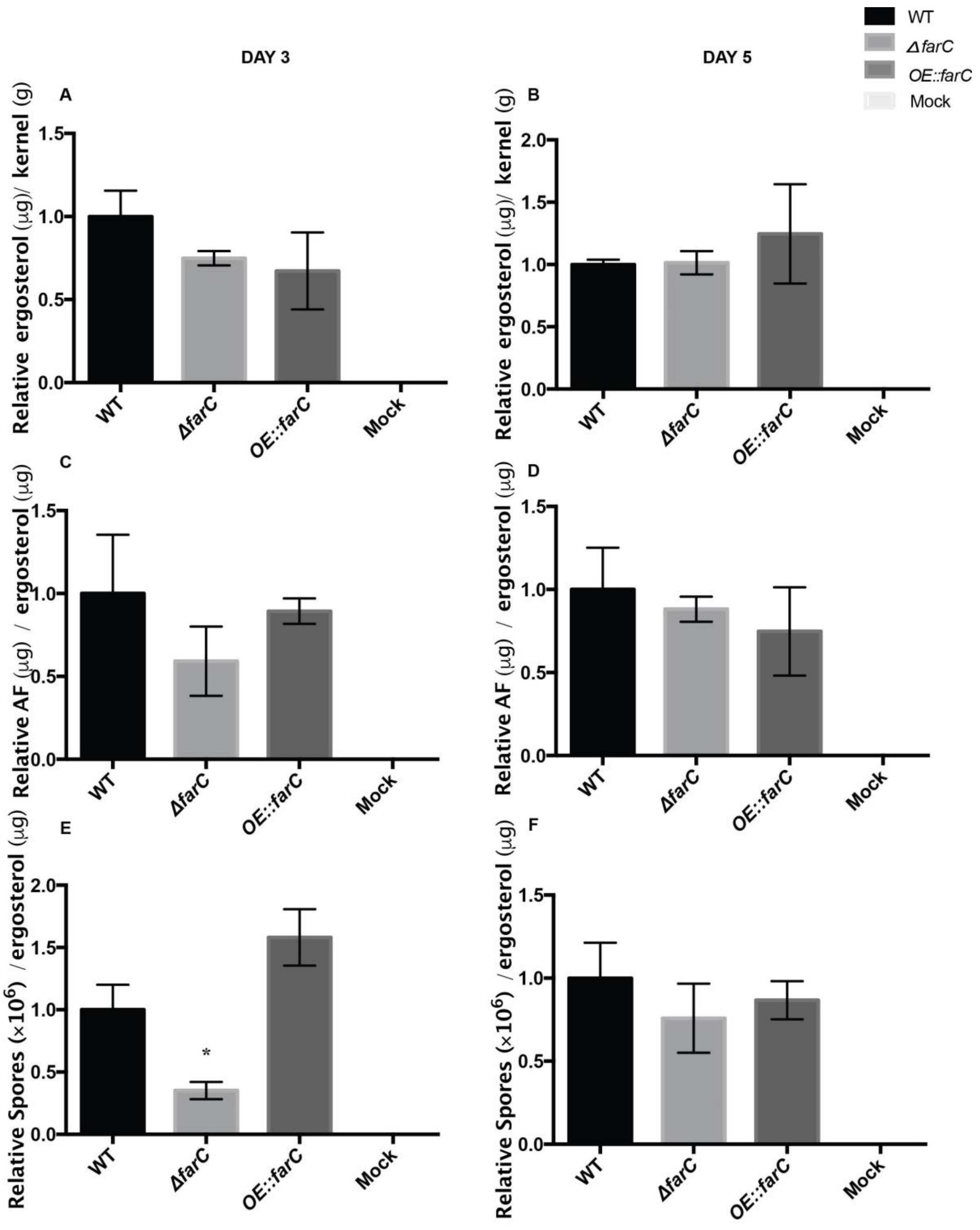

Figure S13 The effects of *farC* on virulence on maize kernels. Maize kernels were infected with *far* mutant strains. Ergosterol was extracted after two (A) and three days (B) and quantified by HPLC. Aflatoxin was also extracted at two (C) and three days (D) and quantified by HPLC. Spores were washed from the kernels after two (E) and three days (F) and counted. For all graphs, wildtype data was

normalized to be 1. The shades of the bars correspond to the legend on the right. Asterisks indicate statistical significance compared to wildtype as determined by a two-tailed Student’s T-test with $*p < 0.05$, $**p < 0.01$, and $***p < 0.001$.
