# Supplementary material for: Characterization of the Far Transcription Factor Family in Aspergillus flavus
Source: G3 (Bethesda). 2016 Aug 16;6(10):3269–81. doi: 10.1534/g3.116.032466 (PMC5068947; doi:10.1534/g3.116.032466)
Supplement: Supplemental Material [file supp_g3.116.032466_TableS1.pdf]

Table S1 All strains used in this study and their genotypes

**Strain**	**Genotype**	**Source**
CA14Δ*ku70*Δ*pyrG*	Δ*nkuA; ΔpyrG*	Chang *et al*. (2010)
TKJA38.6	Δ*farA::pyrG; ΔnkuA; ΔpyrG*	This study
TKJA39.4	Δ*farB::pyrG; ΔnkuA; ΔpyrG*	This study
XL1.1	Δ*farC::pyrG; ΔnkuA; ΔpyrG*	This study
TKJA40.5	*gpdA(p)::farA::pyrG; ΔpyrG*	This study
TKJA41.5	*gpdA(p)::farB::pyrG; ΔpyrG*	This study
XL1.2	*gpdA(p)::farC::pyrG; ΔpyrG*	This study
TKJA13.1	Δ*nkuA::pyrG; ΔpyrG*	Affeldt *et al*. (2014)
