# Supplementary material for: Characterization of the Far Transcription Factor Family in Aspergillus flavus
Source: G3 (Bethesda). 2016 Aug 16;6(10):3269–81. doi: 10.1534/g3.116.032466 (PMC5068947; doi:10.1534/g3.116.032466)
Supplement: Supplemental Material [file supp_g3.116.032466_TableS2.pdf]

Table S2 Oligonucleotide primers used for strain construction and confirmation

No.	Name	Sequence (5' to 3')
1	KS farA 5'fk F	cctccaagtccactgttctg
2	KS farA 5'fk+AfupyrG R	cgaagagggtgaagagcattgtttgaggcacgttgtctatggaaacagcg
3	KS farA 3'fk+AfupyrG F	gtgacgacaatacctcccgacgatacctggctttgaacgtggaagactgg
4	KS farA 3'fk R	taggtggcgttcaaggtgcg
5	KS farB 5'fk F	caatcacagtgtggatctgg
6	KS farB 5'fk+AfupyrG R	cgaagagggtgaagagcattgtttgaggcatagttgctggatctgcgagg
7	KS farB 3'fk+AfupyrG F	gtgacgacaatacctcccgacgatacctggtcgaagccgaacttggattg
8	KS farB 3'fk R	atgagcaaccgggaactgtg
9	XL farC 5'fk F	tccgctcaaagctggagagg
10	XL farC 5'fk+AfupyrG R	cgaagagggtgaagagcattgtttgaggcacgaggaagattgaatgccgc
11	XL farC 3'fk+AfupyrG F	gtgacgacaatacctcccgacgatacctggagtgtgctaacggccctcag
12	XL farC 3'fk R	atcacgccagatttggccgc
13	KS Afu pyrG F	tgcctcaaacaatgctcttc
14	KS Afu pyrG R	ccaggtatcgtcgggaggt
15	KS JP Extended T7 F	cgtaatacgactcactataggg
16	KS JP gpdA(p) fusion R	catggtgatgtctgctcaag
17	XL OEfarA 5’fk F	tggttccttcactgggaggc
18	XL OEfarA 5'fk R	caattcgccctatagtgagtcgtattacgggccaaaaagtatccatgcag
19	XL OEfarA 3'fk F	ctaccccgcttgagcagacatcaccatgatgagcacaacaggagaaaacc
20	XL OEfarA 3'fk R	agattcgaggactcgccgag
21	KS OEfarB 5'fk F	gacgcacctgtcctttgacc
22	KS OEfarB 5'fk R	ccaattcgccctatagtgagtcgtattacgtgtgaagtgtgcggttttag
23	KS OEfarB 3'fk F	agctaccccgcttgagcagacatcaccatgactaatctcactgcctcgcc
24	KS OEfarB 3'fk R	cctggcgcgttgaaagaatg
25	XL OEfarC 5'fk R	ccaattcgccctatagtgagtcgtattacggtctggtctgcttatcgacg
26	XL OEfarC 3'fk F	agctaccccgcttgagcagacatcaccatggcggcattcaatcttcctcg
27	XL OEfarC 3'fk R	tgcaatgggccatgtcttggc
