# Supplementary material for: Characterization of the Far Transcription Factor Family in Aspergillus flavus
Source: G3 (Bethesda). 2016 Aug 16;6(10):3269–81. doi: 10.1534/g3.116.032466 (PMC5068947; doi:10.1534/g3.116.032466)
Supplement: Supplemental Material [file supp_g3.116.032466_TableS3.pdf]

**Table S3 Semi-quantitative RT-PCR oligonucleotide primers and cycling conditions**

| No. | Name | Sequence (5' to 3') | #Cycles | $T_A$ (°C)[a] |
|---|---|---|---|---|
| 1 | XL farA int F | tacatcactcacagagtcgc | 28 | 57 |
| 2 | XL farA int R | agtactgctgcgcaagtacc | | |
| 3 | KS farB int F | ctactggatccttctttcgc | 28 | 57 |
| 4 | KS farB int R | tgggaatgcaggacttcttg | | |
| 5 | KS farC qRT F | tctggagccgcttgaactcg | 28 | 57 |
| 6 | KS farC qRT R | tcgctgacagaaacgtatgg | | |
| 7 | KS ubiD qRT F | aacatccagaaggagtccac | 25 | 55 |
| 8 | KS ubiD qRT R | taccagcgaagatcaaacgc | | |
| 9 | XL pexK qRT F | tcctccgtacgctgcaatac | 25 | 57 |
| 10 | XL pexK qRT R | tccgccattgagacaccacg | | |
| 11 | XL echA qRT F | atggcgcccttgaccttctc | 28 | 57 |
| 12 | XL echA qRT R | tcgaccactcaggcttcttc | | |
| 13 | XL scdA qRT F | aacacactcgtgaacacggc | 25 | 57 |
| 14 | XL scdA qRT R | agaagtttcgcgatggtctg | | |
| 15 | XL foxA qRT F | tcccttggttcaacaccacc | 25 | 57 |
| 16 | XL foxA qRT R | gacacccagagagcacagac | | |
| 17 | XL acuJ qRT F | tctgcaggaaagacttctgg | 25 | 57 |
| 18 | XL acuJ qRT R | aaggcttgtcgtaccagcgg | | |
| 19 | XL eciA qRT F | ttcgggaaatcgacaagcgc | 25 | 57 |
| 20 | XL eciA qRT R | atgcttcttctctcccgaag | | |
| 21 | XL derA qRT F | tgagtgatacgtggaaggac | 25 | 57 |
| 22 | XL derA qRT R | gacttggagtcaaccttgtg | | |
| 23 | XL facA qRT F | tgagaaacaccaggtgactc | 25 | 57 |
| 24 | XL facA qRT R | tccgcaggatacgtcgcatg | | |
| 25 | XL aflR qRT F | agcaccctgtcttccctaa | 32 | 55 |
| 26 | XL aflR qRT R | ctggtcttcctcatccaca | | |
| 27 | XL aflD qRT F | cacttagccatcacggtca | 32 | 55 |
| 28 | XL aflD qRT R | gagttgagatccatccgtg | | |
| 29 | XL fasA qRT F | tcgtattggatcggaggcgg | 28 | 57 |
| 30 | XL fasA qRT R | tgatgcgtatacactggctc | | |
| 31 | XL sdeA qRT F | accgataccgagaaggatcc | 28 | 57 |
| 32 | XL sdeA qRT R | tgcttgagatcataggcgag | | |
| 33 | XL sdeB qRT F | tctatacgggcatcttcgcc | 28 | 57 |
| 34 | XL sdeB qRT R | aagaaaacgcggaggattcc | | |
| 35 | XL odeA qRT F | tcaaccctagcagccctctg | 28 | 57 |
| 36 | XL odeA qRT R | agccttgaagaatcccgtcc | | |

[a]$T_A$: Annealing temperature used for PCR
