# Supplementary material for: Characterization of the Far Transcription Factor Family in Aspergillus flavus
Source: G3 (Bethesda). 2016 Aug 16;6(10):3269–81. doi: 10.1534/g3.116.032466 (PMC5068947; doi:10.1534/g3.116.032466)
Supplement: Supplemental Material [file supp_g3.116.032466_TableS4.pdf]

Table S4 Presence of putative CREA consensus binding sequence 5′-(C/G)YGGRG-3′ in the 5' region of *A. flavus* genes assessed on this study. Positions relative to start codon of 5′-(C/G)YGGRG-3′ were obtained by scanning 1kb upstream of the proposed start codon. Genes were from http://www.ncbi.nlm.nih.gov/ and http://www.aspgd.org

Gene (accession number)	Position relative to start codon (5′-GCGGAG-3′)	Position relative to start codon (5'-CTGGGG-3')
farA (AFL2G_05109)	No motif found	-241
farB (AFLA_012010)	No motif found	No motif found
pexK (AFLA_036410)	-231	-985
echA (AFLA_043610)	No motif found	No motif found
foxA (AFLA_041590)	No motif found	-361
acuJ (AFLA_135240)	-298	-344
eciA (AFLA_055840)	No motif found	-982
derA (AFLA_061230)	-119, -229	No motif found
aflR (AFLA_139360)	No motif found	No motif found
aflD (AFLA_139390)	No motif found	No motif found
