# Supplementary material for: Characterization of the Far Transcription Factor Family in Aspergillus flavus
Source: G3 (Bethesda). 2016 Aug 16;6(10):3269–81. doi: 10.1534/g3.116.032466 (PMC5068947; doi:10.1534/g3.116.032466)
Supplement: Supplemental Material [file supp_g3.116.032466_FigureS2.pdf]

A

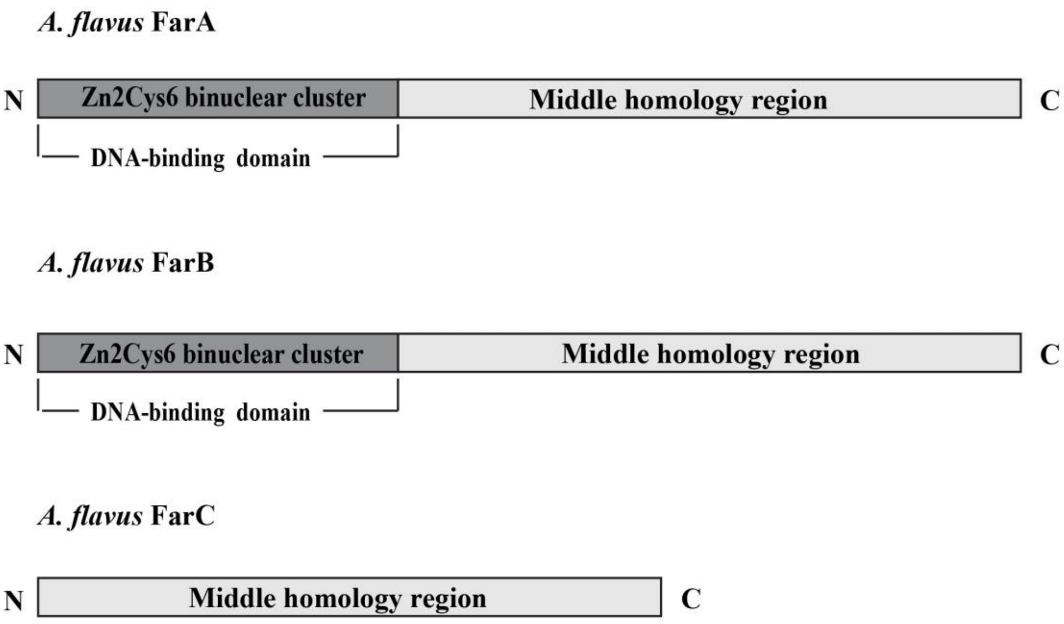

B

```
AFLA_012010_FarB    1 -------MTNLTASPSSSNMAENEAKGKRKASTAGLPANARPVKRRASKACCCRARKVR
AFL2G_05109_FarA    1 MSTTGENHTDSTSRPSPAPSATGSTGTSGITVRAGSNGQMSFRRQRASRACETCHARKVR
AFLA_082910_FarC    1 ------------------------------------------------------------

AFLA_012010_FarB   54 CDVVENGSPCTNCRLDQVDCIVTESKRRKFVHAENMTRLPHSSQNRGLTSSALLCHFPGN
AFL2G_05109_FarA   61 CDAASLGVPCTNCVAFSIECKIPTPKRKKNQTKAKESSGSE-------------------
AFLA_082910_FarC    1 ------------------------------------------------------------

AFLA_012010_FarB  114 HVLRLTTPTTSSLNRPRRLPKTEVFYGDSVNATGF-------RMLPQRRLPSVPIWTRGN
AFL2G_05109_FarA  102 ------------ENPQKETPKDDQSTTDGKDAFGYSSNRMAVDGMPVTSLTESQA-----
AFLA_082910_FarC    1 ------------------------------------------------------------

AFLA_012010_FarB  167 ICHISSVRISLRPGMRLIECKLYTFILISTYIDQSQVSRIGAGPERYRRRMAPNPAVPAT
AFL2G_05109_FarA  145 -A-----Q-------QATQNGAYAQFMKPKFAR-APIK--EAGRVAYLG-----------
AFLA_082910_FarC    1 ------------------------------------------------------------

AFLA_012010_FarB  227 MPLHHVTSQIQQLLDPSFANARSGGIILPDYI---RGLPPRLQKEDIDYLAMKGALTVPD
AFL2G_05109_FarA  178 -----ESSNLSLLVQDRHGTTDVVHYPLPPNIRGSRARLADLDNLELDILHQRGAFLLPP
AFLA_082910_FarC    1 -----------------------------MAEYHSTCHPDHLEPLELEILHRRDAFRLPP

AFLA_012010_FarB  284 VGLRNELLKAYIHYVHTYMPLLDLEDFLQTIAQNDGIRRMSLLLFQAVMFAGTAFVDLKH
AFL2G_05109_FarA  233 KPLCDELVDAYFKWVAPVVPIVNRSRFMRHYRDPK--NPPSLLLLQAILLAGSRVCTNPQ
AFLA_082910_FarC   32 KAVQDTLVEVFFKWVAPILPVVDRDAFLRQYESAE--DSPSILLLQAMLMVASRCSTSEQ

AFLA_012010_FarB  344 LQAAGYSSRKAARKSFFQRARLLYDFDYEVDRISLVQSLLLMTYWYETPDDQK--DTWHW
AFL2G_05109_FarA  291 LMDA-NGSTTPAAMTFYKRAKALYDANYEDDRVTIVQALVLLGWYWEGPEDVTK-NVFYW
AFLA_082910_FarC   90 RS----KEYTVSPRTFYKKAKALYDAGYETNLITVVQAVVLLGAYWEGPDDLTESGIFYW

AFLA_012010_FarB  402 MGVSLSLAHTIGLHRDPGNSRMDVRRQRMWKRIWWSTYTRDRLIALGMRRPMRVKDDDCD
```

```
AFL2G_05109_FarA  349 TRVAMVVAQGSGMHRSVESSQLSKPDKRLWKRIWWTLFTRDRSVAVALGRPIGINTDDSD
AFLA_082910_FarC  146 SRLGIALAQELGLHDSERYTGLQPSERGLRKRIWWTLYTRDRSVAAAFGRPLHINPNYCT

AFLA_012010_FarB  462 VPMLTLDDFEFHPFSPEIVSMVGNSEVLQNVSHQKELALMFIEKAKLCLCVSHVLSAQYS
AFL2G_05109_FarA  409 VGMLTEDDFIEDEIDIA-----------AEYPPDPVHVQFFLQYVKLCEIMGLVLAQQYS
AFLA_082910_FarC  206 VEPLTESDFVEYDGNA------------PSEPTGEVQARFFMEYVKLCQLMDLGLCLNLS

AFLA_012010_FarB  522 VLSHKFGGTMETTMMLVPKKSAAETFEVRRCDQELEDWLAHLPSEIQYAPMAPAKLTEAQ
AFL2G_05109_FarA  458 VASKS---------------RRMNAMDLTHSDMALADWLQNCPKEVCWQRQNH-------
AFLA_082910_FarC  254 ARSTQ----------------DARSAGAAQCELGLNEWLVACPPELHWRQTRH-------

AFLA_012010_FarB  582 EVLHSHRALLKMVYLTTSSALHRPQVLPAMPFPSTDA--ELQDISRNKVRFAAVEITNIA
AFL2G_05109_FarA  496 ---HFWAALLHANYYTTLCLLHRAHMPPASSAPNSYRVEEMAYPSRTIAFQAAGMITSIV
AFLA_082910_FarC  291 ---TFLSAILFSTF----------------------------------------------

AFLA_012010_FarB  640 QDLHALDLTRYFPTTGVTVLLPAVIIHLLDIKSSDPNVRMVSLQRFYQCMRILQRLREIY
AFL2G_05109_FarA  553 ENLQTHQEIRYTPAFIVYSLFSALIMHVYQMRSSVPTVVATCQERINICMQALKDVSKVW
AFLA_082910_FarC      ------------------------------------------------------------

AFLA_012010_FarB  700 ASADFATSFLEAAIRKAGIQLTVAP----QDVQ--------------SRNNCTFDSVRLN
AFL2G_05109_FarA  613 LVAKMVRTLFESILGNKVLEERLQKAAGKRHQRIRHDTAQHQPPRKPDPPKRKFDDMDLG
AFLA_082910_FarC      ------------------------------------------------------------

AFLA_012010_FarB  742 TLT---PPPDSLAQKIPDLTYPKTSGTRLAGEAAEASGFASTPPPSDGSENGSTNNINPH
AFL2G_05109_FarA  673 LPNGGPTPPVSYERSRPQ--TPAVTPSREMGQPGLNVPQGSPTGPPAGNSRGNTRPTTPF
AFLA_082910_FarC      ------------------------------------------------------------

AFLA_012010_FarB  799 YHQDAFAIPNLDSDLSISELMDLANDAEVTQNDFDALINFDDTGAELF---------AAD
AFL2G_05109_FarA  731 --NAQFSLPATPPDLFLVTRTSPNLS-PSLWENFQPDQLFPD-GTAIFPELTSPQQTAVD
AFLA_082910_FarC      ------------------------------------------------------------
AFLA_012010_FarB  850 DGLDLNGNPKGQGYGF-----------NIGTMDNVPDLFGTESKGVGLTGLGNGQLHEDR
AFL2G_05109_FarA  787 PQLQMSSQLQTQGMDQRHMMPHQMSSRGLPGTQGSPEMISNIPPGLGMQGQQPPQVFGME
AFLA_082910_FarC      ------------------------------------------------------------

AFLA_012010_FarB  899 TSTT-----------LGA-------------NEAPRATELDGIADLEAELGLNL------
AFL2G_05109_FarA  847 NQQPWPMAGLEAALHTGVEAASQDDTWSNSSRSGPTAPTTLNVEDWFQFFGINGSFGDLS
AFLA_082910_FarC      ------------------------------------------------------------

AFLA_012010_FarB      ---
AFL2G_05109_FarA  907 TSA
AFLA_082910_FarC      ---
```

Figure S2 Functional domains and multiple sequence alignment of Far proteins in *A. flavus*. (A) These regions contribute to protein-DNA and protein-protein interactions. MHR, fungal-specific middle homology region. (B) Multiple sequence alignment (using Clustal) showing three *A. flavus* Far protein alignment and FarC lacking Zn2Cys6 DNA binding domain.
