# Supplementary material for: Characterization of the Far Transcription Factor Family in Aspergillus flavus
Source: G3 (Bethesda). 2016 Aug 16;6(10):3269–81. doi: 10.1534/g3.116.032466 (PMC5068947; doi:10.1534/g3.116.032466)
Supplement: Supplemental Material [file supp_g3.116.032466_FigureS3.pdf]

A. flavus FarA (AFL2G_05109) protein obtained from AspGD (http://www.aspgd.org):

MSTTGENHTDSTSRPSPAPSATGSTGTSGITVRAGSNGQMSFRRQRASRACETCHARKVRCDAASLGV
PCTNCVAFSIECKIPTPKRKKNQTKAKESSGSEENPQKETPKDDQSTTDGKDAFGYSSNRMAVDGMPVT
SLTESQAAQQATQNGAYAQFMKPKFARAPIKEAGRVAYLGESSNLSLLVQDRHGTTDVVHYPLPPNIRG
SRARLADLDNLELDILHQRGAFLLPPKPLCDELVDAYFKWVAPVVPIVNRSRFMRHYRDPKNPPSLLLLQA
ILLAGSRVCTNPQLMDANGSTTPAAMTFYKRAKALYDANYEDDRVTIVQALVLLGWYWEGPEDVTKNVF
YWTRVAMVVAQGSGMHRSVESSQLSKPDKRLWKRIWWTLFTRDRSVAVALGRPIGINTDDSDVGMLTE
DDFIEDEIDIAAEYPPDPVHVQFFLQYVKLCEIMGLVLAQQYSVASKSRRMNAMDLTHSDMALADWLQNC
PKEVCWQRQNHHFWAALLHANYYTTLCLLHRAHMPPASSAPNSYRVEEMAYPSRTIAFQAAGMITSIVE
NLQTHQEIRYTPAFIVYSLFSALIMHVYQMRSSVPTVVATCQERINICMQALKDVSKVWLVAKMVRTLFESI
LGNKVLEERLQKAAGKRHQRIRHDTAQHQPPRKPDPPKRKFDDMDLGLPNGGPTPPVSYERSRPQTPA
VTPSREMGQPGLNVPQGSPTGPPAGNSRGNTRPTTPFNAQFSLPATPPDLFLVTRTSPNLSPSLWENF
QPDQLFPDGTAIFPELTSPQQTAVDPQLQMSSQLQTQGMDQRHMMPHQMSSRGLPGTQGSPEMISNIP
PGLGMQGQQPPQVFGMENQQPWPMAGLEAALHTGVEAASQDDTWSNSSRSGPTAPTTLNVEDWFQF
FGINGSFGDLSTSA

A. flavus FarB (AFLA_012010) protein obtained from NCBI (http://www.ncbi.nlm.nih.gov/):

MTNLTASPSSSNMAENEAKGKRKASTAGLPANARPVKRRASKACCCCRARKVRCDVVENGSPCTNCRL
DQVDCIVTESKRRKFVHAENMTRLPHSSQNRGLTSSALLCHFPGNHVLRLTTPTTSSLNRPRRLPKTEVF
YGDSVNATGFRMLPQRRLPSVPIWTRGNICHISSVRISLRPGMRLIECKLYTFILISTYIDQSQVSRIGAGPE
RYRRRMAPNPAVPATMPLHHVTSQIQQLLDPSFANARSGGIILPDYIRGLPPRLQKEDIDYLAMKGALTVP
DVGLRNELLKAYIHYVHTYMPLLDLEDFLQTIAQNDGIRRMSLLLFQAVMFAGTAFVDLKHLQAAGYSSRK
AARKSFFQRARLLYDFDYEVDRISLVQSLLLMTYWYETPDDQKDTWHWMGVSLSLAHTIGLHRDPGNSR
MDVRRQRMWKRIWWSTYTRDRLIALGMRRPMRVKDDDCDVPMLTLDDFEFHPFSPEIVSMVGNSEVLQ
NVSHQKELALMFIEKAKLCLCVSHVLSAQYSVLSHKFGGTMETTMMLVPKKSAAETFEVRRCDQELEDW
LAHLPSEIQYAPMAPAKLTEAQEVLHSHRALLKMVYLTTSSALHRPQVLPAMPFPSTDAELQDISRNKVRF
AAVEITNIAQDLHALDLTRYFPTTGVTVLLPAVIIHLLDIKSSDPNVRMVSLQRFYQCMRILQRLREIYASAD
FATSFLEAAIRKAGIQLTVAPQDVQSRNNCTFDSVRLNTLTPPPDSLAQKIPDLTYPKTSGTRLAGEAAEA

SGFASTPPPSDGSENGSTNNINPHYHQDAFAIPNLDSDLSISELMDLANDAEVTQNDFDALINFDDTGAEL

FAADDGLDLNGNPKGQGYGFNIGTMDNVPDLFGTESKGVGLTGLGNGQLHEDRTSTTLGANEAPRATE

LDGIADLEAELGLNL

A. flavus FarC (AFLA_082910) protein obtained from NCBI (http://www.ncbi.nlm.nih.gov/):

MAEYHSTCHPDHLEPLELEILHRRDAFRLPPKAVQDTLVEVFFKWVAPILPVVDRDAFLRQYESAEDSPSI

LLLQAMLMVASRCSTSEQRSKEYTVSPRTFYKKAKALYDAGYETNLITVVQAVVLLGAYWEGPDDLTESG

IFYWSRLGIALAQELGLHDSERYTGLQPSERGLRKRIWWTLYTRDRSVAAAFGRPLHINPNYCTVEPLTE

SDFVEYDGNAPSEPTGEVQARFFMEYVKLCQLMDLGLCLNLSARSTQDARSAGAAQCELGLNEWLVAC

PPELHWRQTRHTFLSAILFSTF

Figure S3 Amino acid sequence of *A. flavus* FarB and FarC protein. C6 zinc finger consensus amino acid sequence was labeled with red. FarA protein sequence was obtained from AspGD (http://www.aspgd.org). FarB and FarC protein sequences were obtained from Genbank (http://www.ncbi.nlm.nih.gov/).
