# Supplementary material for: Characterization of the Far Transcription Factor Family in Aspergillus flavus
Source: G3 (Bethesda). 2016 Aug 16;6(10):3269–81. doi: 10.1534/g3.116.032466 (PMC5068947; doi:10.1534/g3.116.032466)
Supplement: Supplemental Material [file supp_g3.116.032466_FigureS4.pdf]

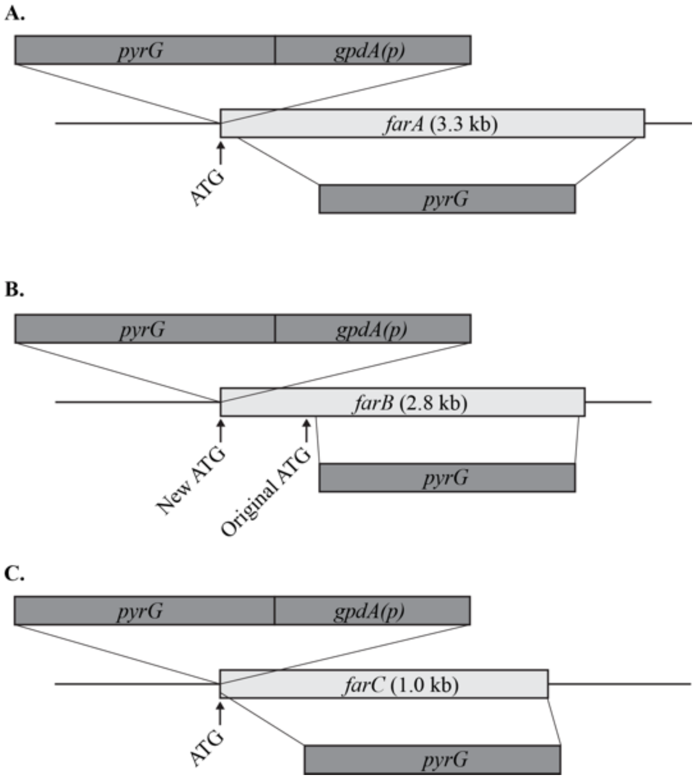

Figure S4 *farA, farB* and *farC* mutant constructs. (A) The gene encoding *farA* is 3.3 kb in length. An overexpression cassette containing a *pyrG* marker and the constitutively active *gpdA* promoter was inserted immediately upstream of the ATG site to generate an overexpression mutant. *farA* was disrupted with *pyrG*. (B) Alignment of FarB with FarB orthologs indicated that the true start site was 657 bp upstream of the annotated start site, extending *farB* to 2.8 kb in length. The same overexpression cassette used for *farA* was inserted immediately upstream of the new ATG site to generate an overexpression mutant. *farB* was also disrupted with *pyrG*. (C) The gene encoding *farC* is 1.0 kb in length. An overexpression cassette containing a *pyrG* marker and the constitutively active *gpdA* promoter was inserted immediately upstream of the ATG site to generate an overexpression mutant. *farC* was disrupted with *pyrG*.
