# Supplementary material for: Characterization of the Far Transcription Factor Family in Aspergillus flavus
Source: G3 (Bethesda). 2016 Aug 16;6(10):3269–81. doi: 10.1534/g3.116.032466 (PMC5068947; doi:10.1534/g3.116.032466)
Supplement: Supplemental Material [file supp_g3.116.032466_FigureS5.pdf]

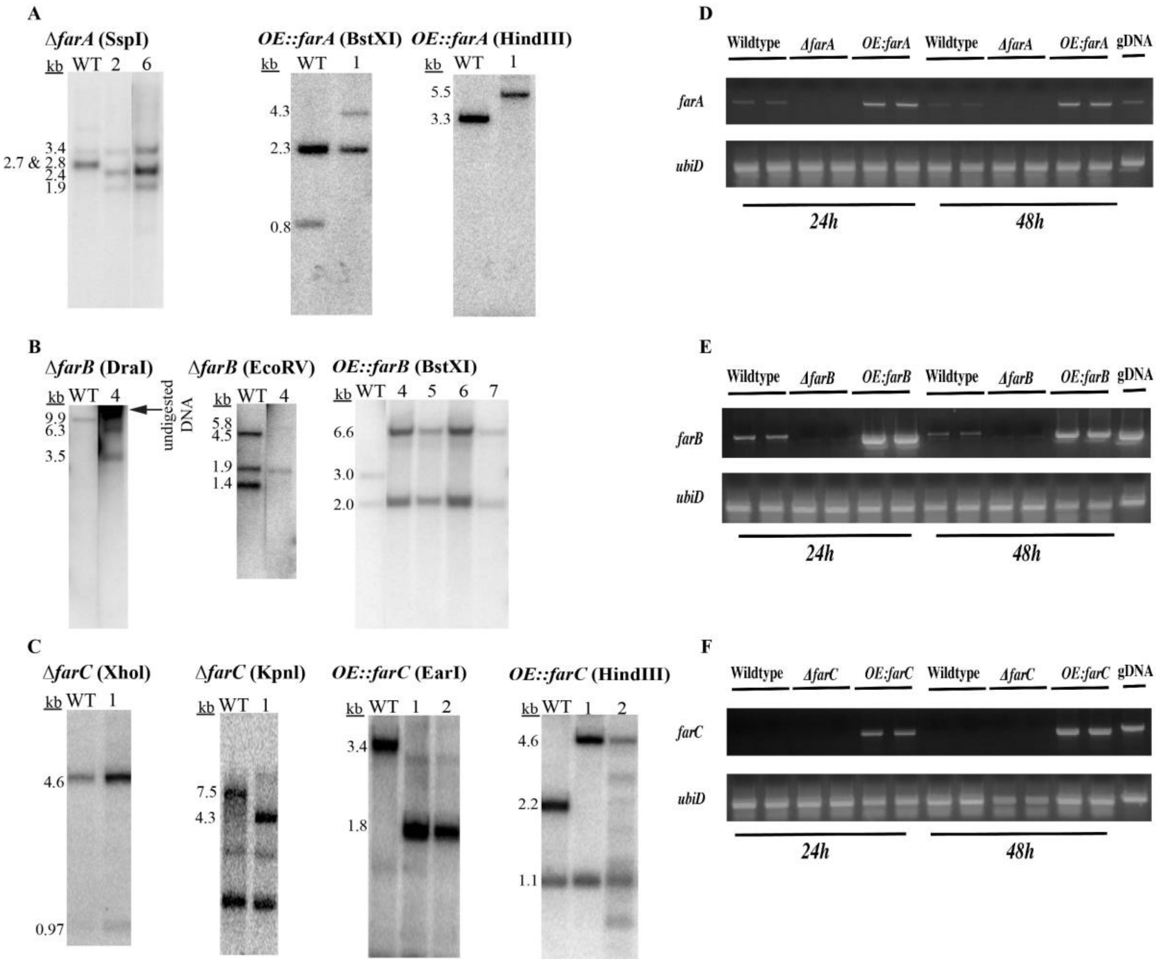

Figure S5 Strain confirmation. (A) Potential Δ*farA* transformants were digested with SspI and *OE::farA* with BstXI and HindIII, respectively, and probed by Southern blot. Expected sizes for the SspI digest were as follows. WT: 2.7, 2.8, and 3.4 kb; Δ*farA*: 1.9, 2.4, and 3.4 kb. Expected sizes for the BstXI digest were as follows. WT: 2.3 and 0.8 kb; *OE::farA*: 2.3 and 4.3 kb. Expected sizes for the BstXI digest were as follows. WT: 3.3 kb; *OE::farA*: 5.5 kb. **(B)** Potential Δ*farB* and *OE::farB* transformants were digested with DraI. EcoRV, and BstXI and probed by Southern blot. Expected sizes for the DraI digest were as follows. WT: 9.9 kb; Δ*farB*: 3.5 and 6.3 kb. Expected sizes for the EcoRV digest were as follows. WT: 1.4, 1.9, and 4.5 kb; Δ*farB*: 1.9 and 5.8 kb (faint). Expected sizes for the BstXI digest were as follows. WT: 2.0 and 3.0 kb; *OE::farB*: 2.0 and 6.6 kb. **(C)** Potential Δ*farC* transformants were digested with XhoI and KpnI, and probed with a 1.5 kb fragment of 5'-UTR and a 1.5 kb 3'-UTR respectively to identify Δ*farC* mutants based on a size difference. Expected sizes for the XhoI digest were as follows. WT: 4.6 kb; Δ*farC*: 0.97 and 4.6

kb; OE*::farC*: 4.6 kb and 5.7 kb . Expected sizes for the KpnI digest were as follows. WT: 7.5 kb; ∆*farC*: 4.3 kb. Potential *OE::farC* transformants were digested with EarI and HindIII and probed with a 1.5 kb fragment of 5'-UTR and a 1.5 kb 3'-UTR respectively. Transformant 1 was chosen as putative ∆*farC* mutants. Expected sizes for the EarI digest were as follows. WT: 3.4 kb; OE*::farC*: 1.8 kb. Expected sizes for the HindIII digest were as follows. WT: 1.1 kb and 2.2 kb; OE*::farC*: 1.1 kb and 4.6 kb. **(D; E; F)** Respective trains were grown in liquid GMM and RNA was extracted at 24 and 48 hours post inoculation and converted to cDNA. Semi-quantitative PCR was carried out, with wildtype gDNA as the positive control.
