# Supplementary material for: Characterization of the Far Transcription Factor Family in Aspergillus flavus
Source: G3 (Bethesda). 2016 Aug 16;6(10):3269–81. doi: 10.1534/g3.116.032466 (PMC5068947; doi:10.1534/g3.116.032466)
Supplement: Supplemental Material [file supp_g3.116.032466_FigureS6.pdf]

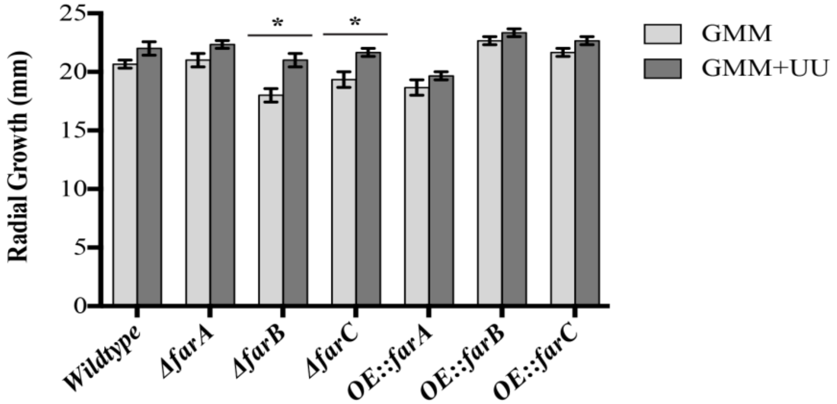

Figure S6 *farA, farB* and *farC* mutant marker gene effects. Strains were grown on media with and without the addition of uridine and uracil (UU) and radial growth was measured to determine whether growth defects were due to the *pyrG* marker gene.
