# Supplementary material for: Characterization of the Far Transcription Factor Family in Aspergillus flavus
Source: G3 (Bethesda). 2016 Aug 16;6(10):3269–81. doi: 10.1534/g3.116.032466 (PMC5068947; doi:10.1534/g3.116.032466)
Supplement: Supplemental Material [file supp_g3.116.032466_FigureS7.pdf]

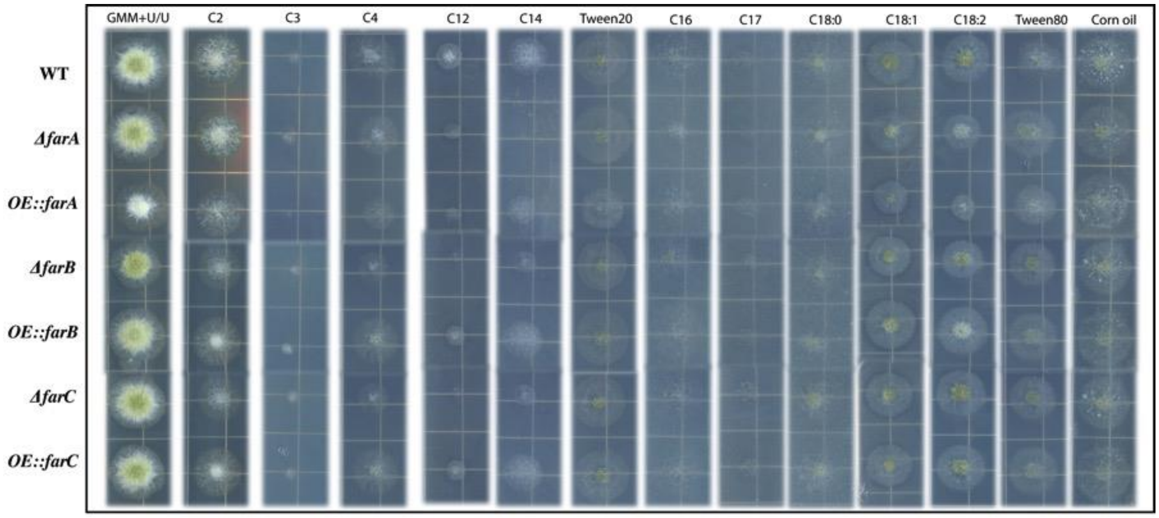

Figure S7 Growth of *far* mutants and the wildtype on fatty acids as sole carbon sources. The following carbon sources were added to minal medium (UU+1% tergitol+70.6 mM ammonium chloride) with glucose (1%); sodium acetate (C2) and propionic acid (C3) (50 mM); sodium butyrate (C4) (10 mM); lauric (C12) (2.5mM); myristic (C14) (2.5mM); palmitic (C16) (2.5mM); heptadecanoic (C17) (2.5mM); stearic (C18:0) (2.5mM); oleate (C18:1) (2.5mM); linoleic (C18:2) (2.5mM) and erucic acids (C22:1) (2.5 mM); Tween 20 and Tween 80 (0.1%); Corn oil (1.0%). Plates were incubated at 29°C for 3 days.
