# Supplementary material for: Characterization of the Far Transcription Factor Family in Aspergillus flavus
Source: G3 (Bethesda). 2016 Aug 16;6(10):3269–81. doi: 10.1534/g3.116.032466 (PMC5068947; doi:10.1534/g3.116.032466)
Supplement: Supplemental Material [file supp_g3.116.032466_FigureS8.pdf]

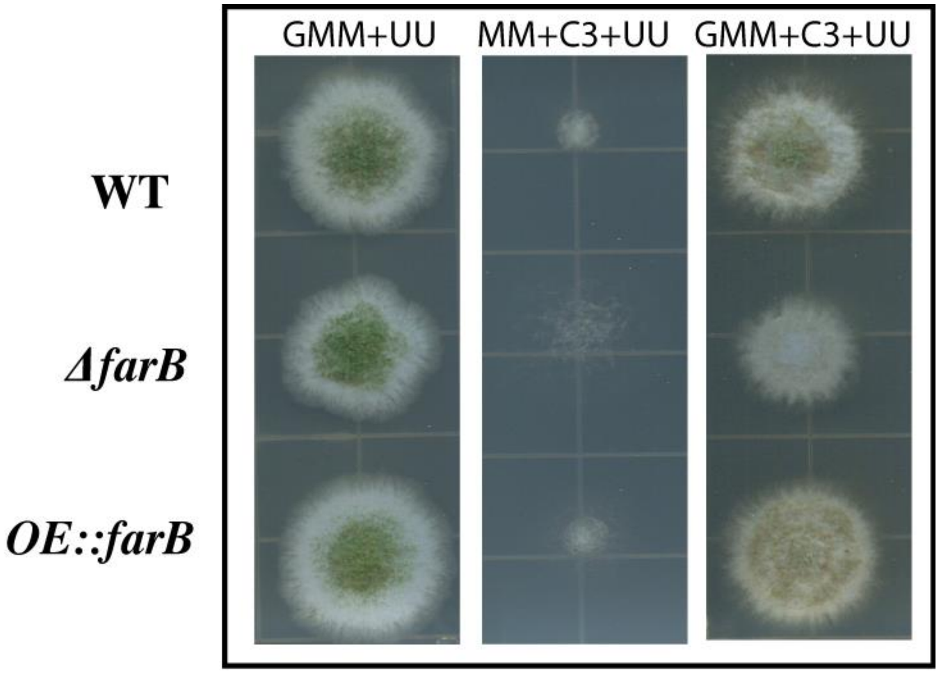

Figure S8 Growth test of *farB* mutants treated with propionic acid (C3). Strains were grown on minimal medium (MM) + UU with glucose or propionic acid (C3) as sole carbon source.
