# Supplementary material for: Characterization of the Far Transcription Factor Family in Aspergillus flavus
Source: G3 (Bethesda). 2016 Aug 16;6(10):3269–81. doi: 10.1534/g3.116.032466 (PMC5068947; doi:10.1534/g3.116.032466)
Supplement: Supplemental Material [file supp_g3.116.032466_FigureS9.pdf]

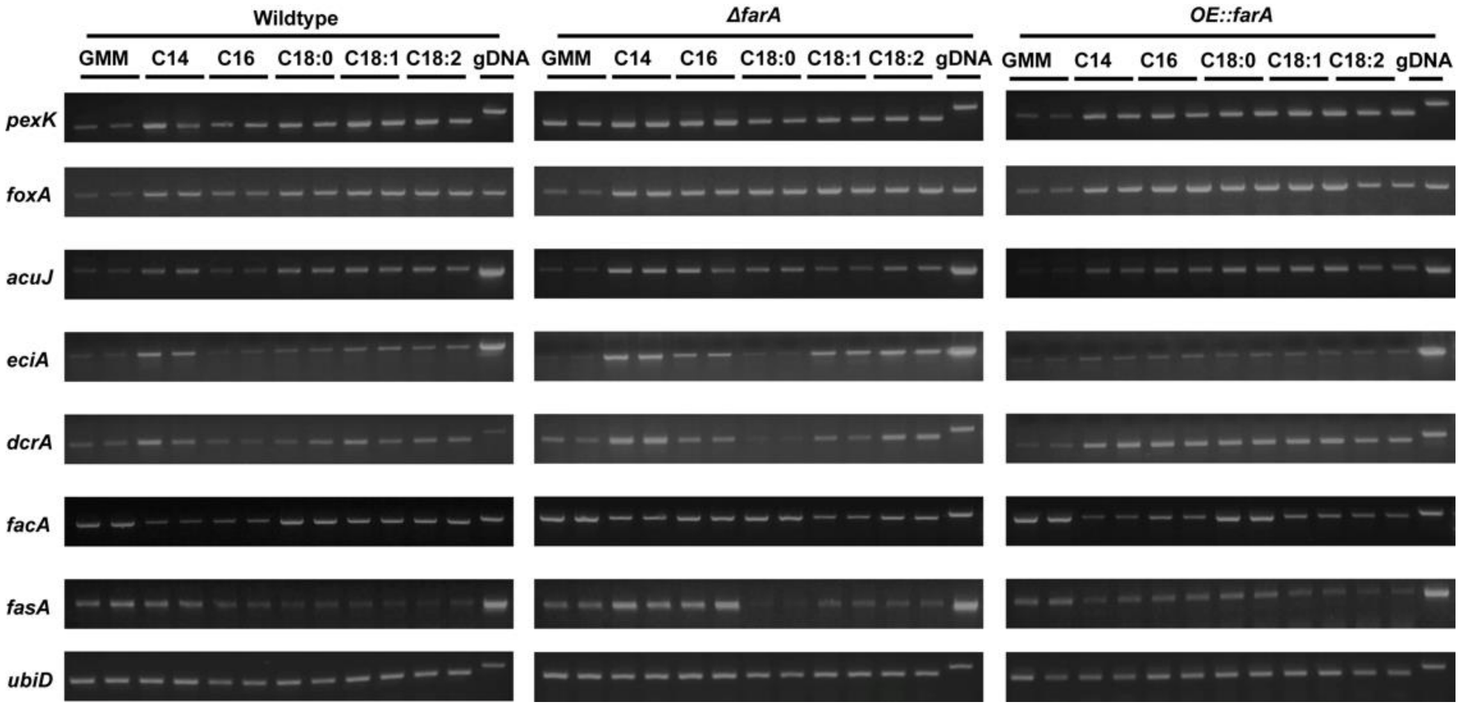


**Figure S9** Expression of genes on *farA* mutants under different carbon sources. Strains were grown for 24 hours in liquid GMM($NH_4^+$)+UU, then transferred to fresh containing minimal medium (UU+1% tergitol+70.6 mM ammonium chloride) with 1% glucose, 2.5 mM myristic acid (C14), 2.5 mM palmitic acid (C16), 2.5 mM stearic acid (C18:0), 2.5 mM oleic acid (C18:1) and 2.5 mM linoleic acid (C18:2). After four hours, mycelia were collected. RNA was extracted and converted to cDNA, and semi-quantitative RT-PCR was performed. Expression of housekeeping gene *ubiD,* which encodes ubiquitin, was included as a control.
